# Cancer therapeutic approach based on conformational stabilization of mutant p53 protein by small peptides

**DOI:** 10.18632/oncotarget.7857

**Published:** 2016-03-02

**Authors:** Perry Tal, Shay Eizenberger, Elad Cohen, Naomi Goldfinger, Shmuel Pietrokovski, Moshe Oren, Varda Rotter

**Affiliations:** ^1^ Department of Molecular Cell Biology, The Weizmann Institute of Science, Rehovot, Israel; ^2^ Department of Molecular Genetics, The Weizmann Institute of Science, Rehovot, Israel

**Keywords:** p53, reactivation, peptides, conformation, pre-clinical

## Abstract

The p53 tumor suppressor serves as a major barrier against malignant transformation. Over 50% of tumors inactivate p53 by point mutations in its DNA binding domain. Most mutations destabilize p53 protein folding, causing its partial denaturation at physiological temperature. Thus a high proportion of human tumors overexpress a potential potent tumor suppressor in a non-functional, misfolded form. The equilibrium between the properly folded and misfolded states of p53 may be affected by molecules that interact with p53, stabilizing its native folding and restoring wild type p53 activity to cancer cells. To select for mutant p53 (mutp53) reactivating peptides, we adopted the phage display technology, allowing interactions between mutp53 and random peptide libraries presented on phages and enriching for phage that favor the correctly folded p53 conformation. We obtained a large database of potential reactivating peptides. Lead peptides were synthesized and analyzed for their ability to restore proper p53 folding and activity. Remarkably, many enriched peptides corresponded to known p53-binding proteins, including RAD9. Importantly, lead peptides elicited dramatic regression of aggressive tumors in mouse xenograft models. Such peptides might serve as novel agents for human cancer therapy.

## INTRODUCTION

The p53 tumor suppressor acts as a major barrier against cancer progression. The wild type (WT) p53 protein responds to various types of cellular stress and triggers cell cycle arrest, apoptosis, or senescence [1, 2]. This is achieved in part through transactivation of specific target genes carrying p53 DNA binding motifs [3-5]. However, almost all human cancers exhibit an impaired p53 pathway [6]. Mutation of p53 is considered a critical step in the malignant transformation process, and over 50% of cancer cases carry mutations in the *TP53* gene, encoding p53 [7]. Most of these mutations are missense point mutations that target the DNA-binding core domain (DBD) [8], thereby abolishing specific DNA binding of p53, preventing p53-dependent transcription, and abrogating p53-mediated tumor suppression. Several compelling reasons make mutant p53 (mutp53) an appealing target for cancer therapy; in particular, the exceptionally high frequency of p53 mutations in human tumors of diverse types makes p53 unique among genes involved in tumor development [9, 10].

Structural studies have revealed that many tumor-derived missense mutations in the p53 DBD produce a common effect: destabilization of core domain folding at physiological temperature [11, 12]. This destabilization is reversible since some mutants can revert to WT conformation and bind DNA at reduced temperatures [13].

Mutp53 proteins accumulate to high levels in tumor cells, partly due to their inability to induce the expression of p53's main negative regulator, Mdm2 [14, 15]. Moreover, many p53 activating stress signals (hypoxia, genomic instability and oncogene activation) are strongly and constitutively induced in cancer cells. However, most p53 downstream effectors are not impaired, due to lack of selective pressure for their inactivation once tumor cells have incapacitated p53 itself. Therefore, restoration of p53's WT conformation is expected to exert major effects in cancer cells, due to high p53 protein levels and persistent stress signals [16]. Reactivation of p53 has been recently demonstrated as effective and specific for the elimination of tumors [17]: p53ER^TAM^ knock-in (KI) mice reproduce a classical p53 knockout phenotype with a high incidence of spontaneous tumors. However, systemic administration of 4-OHT to these mice rapidly restores p53 functions in all tissues. Notably, while such restoration is well tolerated in normal tissues and produces no visible toxic effects, in irradiated cells or in tumor cells it leads to augmented p53 activation, unleashing its growth suppressor and apoptotic functions [18].

Thus, more than half of all human tumors overexpress a latent, potentially highly potent tumor suppressor [19]. A molecule that favors the proper folding of mutp53 and restores WT functions in tumors might serve as an efficient and specific anticancer drug [20].

Mutations in the p53 DBD can be classified into two major categories. Several residues, including 120, 241, 248, 273, 276, 277 and 280 are in direct contact with DNA; mutations in these residues weaken the interaction with DNA [21] but sometimes cause only a minor destabilization of the protein conformation [22], and are thus considered DNA contact mutants. Most other cancer-associated mutations, however, affect markedly the folding of the p53 protein, and are considered conformational mutants. Crystallography, NMR studies and quantitative assessment of folding and DNA-binding properties of DBD mutant proteins have revealed that the major effect of conformational mutations is destabilization of the secondary structure of the DBD, lowering of the melting temperature by 5-10°C; this is sufficient to tip the balance towards the misfolded state at physiological temperature [13].

To enable its multiple functions, p53 has evolved into a dynamic and flexible protein [23]. An accepted simplified model suggests that p53 can assume either a WT transcriptionally active conformation or a mutant, misfolded transcriptionally inactive conformation. The two conformational states of p53 can be distinguished by specific antibodies [24]. The mutant-specific PAb240 antibody binds residues 212-217 in the DBD, a region inaccessible in the WT conformation but exposed in denatured conformation [25]. The PAb1620 antibody recognizes a conformational, nonlinear epitope in the p53 DBD, composed of two distinct regions and including residues R156, L206, R209 and N210. The WT p53 protein is folded in a way that holds its loops in close proximity to each other [26], forming the complete epitope recognized by PAb1620. When p53 is misfolded as a result of mutation, high temperature or other drivers of denaturation, these two loops move apart, destroying the PAb1620 epitope. Lack of a rigid structure may result in a number of p53 conformers displaying different activity and conformational state, depending on the type of stress and cellular context [27, 28]. Thus, under specific cellular conditions, genetically mutant p53 may acquire a wild type conformation, presumably through interaction with molecular chaperones, as exemplified in embryonic stem cells [29]. Likewise, wild type p53 may assume a mutant-like conformation as a consequence of particular cancer-associated aberrations [30] Thus, the defect in folding produced by a single amino acid substitution is potentially reversible. Indeed, some p53 mutants maintain residual DNA binding ability; mutants that fail to bind DNA at 37°C still bind at sub-physiological temperatures [31] and activate transcription from a p53-responsive promoter [32]. In addition, the mutant DBD proteins R245S, R282W, V143A and others retain residual (30-60%) DNA-binding activity at 20°C [22].

Structural studies have shown that the extent of misfolding varies among mutants. However, there is no defined alternative conformer but rather a partial denaturation. This suggests that a small molecule reactivation approach to reverse the effect of p53 mutations could be applicable to a wide range of mutp53 isoforms. Another important prediction derived from structural studies is that a ligand that binds to the sometimes very small, properly folded fraction of the protein, is expected to shift the equilibrium towards the native fold by mass action [33].

p53-based cancer therapy has been in the focus of research for quite a time, as restoration of the p53 pathway is likely to have substantial clinical benefits. The approaches taken (reviewed in [34-36]) can be divided into four major groups. The first group is gene therapy, involving introduction of a WTp53 gene into cancer cells, as exemplified by Gendicine (Ad-p53), the first gene therapy product approved for clinical use in humans (China, 2003). No close correlation between p53 mutation status of the tumor cells and response to Ad-p53 treatment was found [37]. The second group is based on inhibition of p53-Mdm2 interactions in cancer cells expressing WTp53 and high Mdm2, e.g by RITA, Nutlin, BDA and MI219. The third group includes molecules that activate proteins either upstream or downstream of p53, including Tenovin-1 and miR-34a. The fourth group consists of molecules that reactivate mutp53, e.g PRIMA-1, MIRA-1, Elipticin, CDB3, WR1065, NSC319726, p53R3 and CP-31398. Most of the above approaches were developed either by rational design or screening of small molecule chemical libraries. The use of rational design often yields molecules like CDB3, with a modest phenotypic effect and an intermediate conformational state of p53 [38]. Screening of chemical libraries has its limitations too; chemical libraries usually have relatively low complexity (10^3^-10^5^ molecules), their screening requires individual assessment of each molecule, and it results at times in molecules having low specificity to the target and high non-specific toxic effects, like CP-31398 [39]. Two small molecules that rescue p53 function in cancer cells, PRIMA-1 and MIRA-1, were identified using cell-based screening assays [40, 41], and were shown to activate mutp53 through binding free cysteine residues.

We now describe the use of phage display to select mutp53-reactivating peptides. Phage peptide display libraries have a much higher complexity than chemical libraries [42]. The selection process is based on binding of peptides to an immobilized target, elution and amplification and finally identification by sequencing, enabling screening of high numbers of molecules in a short time. We developed a procedure combining different selection strategies, different peptide libraries and deep sequencing of selected pools. Lead peptides stabilize the correct conformation of mutp53, endow mutp53 with WTp53-like activities *in vitro* and in live cells, and cause regression of mutp53-bearing tumors in several xenograft models.

## RESULTS

### Calibration of experimental conditions

**Figure 1:**
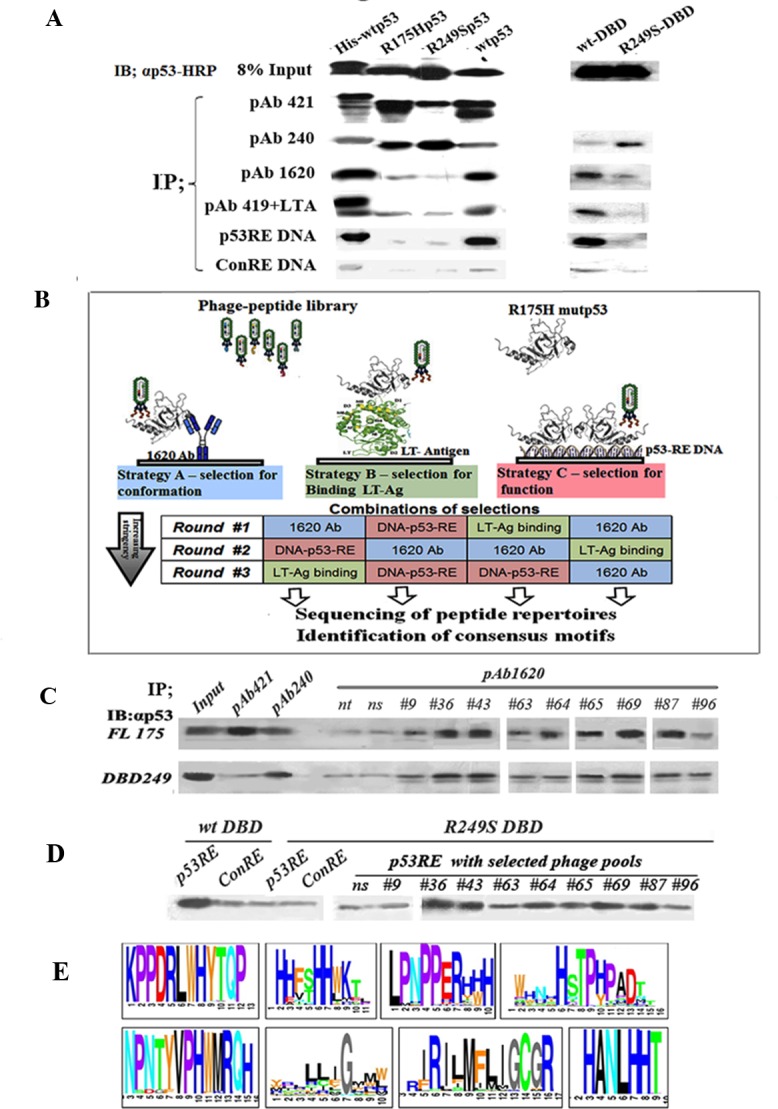
Outline of experimental rationale, calibration of conditions **A.** IP-Western analysis of the binding of WTp53 and mutp53 to various markers distinguishing between WT and mutp53 conformations. 50ng of each purified protein was subjected for immunoprecipitation with a subset of different antibodies and proteins shown to bind differentially WTp53 or mutp53: PAb421 (binds both WT and mutant), PAb1620 (WT specific), PAb240 (mutant specific), PAb419+LTag (WT specific), biotinilated-p53RE DNA oligo (WT specific), and a control oligo mutated at two bases. Immunoprecipitated material was subjected to Western blotting using αp53-HRP as second antibody. **B.** Schematic diagram representing the protocol for identification, screening and selection of mutp53 reactivating peptides. The protocol consists of various selection strategies, at increasing stringencies, for screening and identifying mutp53 reactivating peptides, by utilizing phage display. Strategy A (left): Conformation-based selection: selection of peptides presented by a phage, which can bind a mutp53 protein bound to immobilized WTp53 conformation-specific antibody (PAb1620), thereby enabling selection of a bound phage capable of stabilizing WTp53 conformation. Strategy B (middle): selection according to binding: selection of peptides, which can bind a mutp53 protein bound to immobilized LT-antigen. Strategy C (right): selection according to function: selection of peptides binding to immobilized WTp53-p53RE DNA complex. **C.** Western blot analysis of IP with PAb1620 antibody of purified either p53^R175H^ (upper panel) or p53^R249S^-DBD (lower panel) in the presence of selected phage pools. Non-selected phage (ns) and no phage (nt) were used as controls. Incubation was for 3 hours at 4°C. Bound p53 in the immunoprecipitate (IP) was analyzed by Western blot using antibody against p53 (αp53). “In” stands for 10% of the IP input material, loaded directly on the gel. **D.** Western blot analysis of IP experiments of streptavidin-coated beads bound either to p53RE-DNA or control-RE-DNA oligonucleotides labeled with biotin were incubated with purified WTp53-DBD or mutant p53^R249S^-DBD in the presence of phage selected by phage display. Non selected phage (ns) were used as control. Incubation was for 3 hours at 4°C. **E.** A schematic illustration of several consensus peptide motifs identified as described.

We employed the baculovirus expression system to express p53 protein in SF9 insect cells. The following p53 variants were expressed: His-tagged WTp53, mutp53^R175H^, mutp53^R249S^ and the temperature sensitive (ts) mutant p53^V143A^. In addition, we employed the DBDs of WTp53 and of p53^R249S^ produced in E.coli. To determine p53 conformation and function, proteins were purified (see Supplementary Data) and subjected to immunoprecipitation with a subset of different antibodies and proteins that bind differentially either to WTp53 or to misfolded mutp53. PAb421 binds both WT and mutant p53, PAb1620 is WT-specific, PAb240 is mutant-specific. We also used the PAb419 monoclonal antibody in combination with SV40 large T-antigen (LTag), which binds specifically to WTp53. In addition, we employed a biotinylated p53 response element (p53RE) DNA oligo (WT-specific) and a control oligo mutated at two bases. Immunoprecipitated material was subjected to Western blot analysis using αp53-HRP antibody. As seen in Figure 1A and Figure S1A, WTp53 bound preferentially to PAb1620, p53RE DNA and LTag but not to PAb240; the average PAb1620/PAb240 ratio was 6:1 In contrast, the mutp53 isoforms p53^R175H^ and p53^R249S^ bound preferentially to PAb240 but not to the other interactors, with an average PAb1620/PAb240 ratio of 0.25. These results confirm that the p53 proteins expressed in SF9 cells indeed maintained their expected protein folding states. Subsequently, fine tuning of the assay conditions was performed in order to reduce the relatively high residual binding of mutp53^R175H^ to PAb1620 (Figure S1B).

### Selection of peptides

To identify peptides that stabilize mutp53 in a WT-like functional conformation, we employed a 3-component system (Figure 1B). In the prototypic system, PAb1620 cross-linked to beads was incubated in solution with the phage library and purified recombinant mutp53. In principle, mutp53 should not bind PAb1620. However, when it encounters a peptide that stabilizes its WT conformation, mutp53 is expected to regain PAb1620 binding. This should lead to formation of an immobilized PAb1620-mutp53-phage complex, from which the phage particles can be recovered. A major shortcoming of phage display technology is the abundance of false positive phage that bind non-specifically to elements within the experimental system [43]. Indeed, we observed that consecutive selection cycles with PAb1620 alone resulted in the majority of phage particles binding directly to the antibody instead of through a p53 complex (Table S1A, B). We therefore sought to reduce false positive binders by using different selection/elution strategies in consecutive panning cycles [44]. This was achieved by replacing PAb1620 with either immobilized short DNA that is bound specifically by WTp53 (p53 responsive element, p53RE) or SV40 large T antigen (LT-Ag), which also binds preferentially to the WT conformation of p53. We performed alternating cycles of phage selection, using a different immobilized platform (PAb1620, p53RE DNA or SV40 LT-Ag) at each step, and obtained a marked reduction in false positive binders (Table S1C). Importantly, since each platform selects for a different, complementary trait of WTp53, such strategy is expected to greatly increase the likelihood of selecting peptides that will eventually have biological impact.

We used in parallel 2 commercial random peptide phage display libraries (New England Biolabs): a linear random heptapeptide (PhD-7) library and a dodecapeptide (PhD-12) library. Table S2 shows the different selection routes taken, as well as the phage titers after each selection round. After 3 cycles of selection we obtained over 60 different pools (sub-libraries) greatly enriched for mutp53-reactivating phage (Table S2), which were then subjected to DNA sequencing.

### Validation of phage pool effects on mutp53

To determine whether our selection protocol was effective, we examined the ability of phage pools obtained after 3 cycles of selection to induce the binding of either full length p5^3R175H^ or recombinant p53^S249R^ DBD (249DBD) to PAb1620. As seen in Figure 1C, some selected phage pools indeed elicited increased binding of mutp53 to PAb1620, relative to no phage or non-specific naive phage (ns).

To examine the effect of selected phage pools on the binding of mutp53 to p53 consensus DNA, we employed streptavidin-coated beads to immobilize biotinylated p53RE oligonucleotides or control oligonucleotides mutated in key bases crucial for p53 binding (Con-RE). The p53RE or Con-RE beads were incubated with either WTp53 DBD or mutant p53^R249S^-DBD together with phage pools obtained after 3 cycles of selection. As expected, the wtp53-DBD bound to the p53RE better than to the Con-RE (Figure 1D). The p53^R249S^-DBD did not bind to the p53RE, consistent with its known loss of sequence-specific DNA binding ability. Importantly, several selected phage pools were capable of inducing the binding of mutp53 to the p53RE, demonstrating that they are indeed capable of restoring the lost DNA binding activity of mutp53.

### Deep sequencing of selected phage pools

We next subjected the selected phage pools to next-generation sequencing; DNA was isolated from bacteria infected with 8 different phage pools, and DNA segments coding for the selected peptides were PCR-amplified with primers corresponding to phage sequences flanking the inserted peptide sequences.

The deep sequencing yielded a very large database of 36 million reads. 95% of the sequences contained the correct PCR primers, indicating technical validity of the experiment.

After filtering out irrelevant sequences, our database contained 10^7^ sequences in total. Table 1 shows the top list of deduced peptide sequences. The sequences are divided by their two libraries of origin, namely 12aa and 7aa. #Reads indicates the number of times the particular sequence appears in the database, indicating its extent of enrichment; sequences are sorted in descending order according to the number of reads. Since the bioinformatics analysis was performed on the DNA sequences and because of the genetic code degeneracy, some peptide sequences appear in Table 1 multiple times. #Repeats shows the number of non-identical DNA sequences in the database that code for the same peptide sequence, indicating independent selection of different phage clones.

**Table 1:** Sequences obtained from deep sequencing of selected phage pools

12aa Library			7aa Library		
#Reads	Sequence	#Repeats	#Reads	Sequence	#Repeats
553571	**KPPDRLWHYTQP**	120	194006	**HFSHHLK**	108
71970	**NPNTYVPHWMRQ**	54	149576	**LPNPPER**	81
68333	**ATLPFVTDRQGW**	42	119076	**LHSKTLV**	66
10943	**LRCLLLLIGRVG**	18	96985	**HQVHTHQ**	54
10914	**EFHSFYTARQTG**	11	93473	**KPDSPRV**	60
8643	**NHPWQFPNRWTV**	7	85894	**HEVTHHW**	21
8622	**AILTLILRRVIL**	3	79729	**KPDSPRV**	12
7072	**GAMHLPWHMGTL**	8	76099	**TPPLTLI**	18
6427	**IRILMFLIGCGR**	1	73014	**HTIHPST**	6
5311	**SDGFVPHFKRQH**	4	64810	**HPWTHHQ**	48
1502	**SEFPRSWDMETN**	4	51964	**SAASDLR**	40
1408	**HNHAHSQHTPQH**	8	43941	**SPLQSLK**	33
1362	**WNHHHSTPHPAH**	1	39254	**RPTQVLH**	27
1320	**LRSYAFSFVPPF**	3	39167	**TRILLIGR**	24
1218	**KDLPFYSHLSRQ**	7	36985	**WTLSNYL**	30
1391	**SFILFIRRLGRL**	8	23377	**IRILMFS**	6
1294	**LHNKHRPEPDSG**	6	17441	**VPHIHEF**	12
810	**RLIVRILKLPSP**	3	6945	**HDTHNAH**	10
633	**SILTLRLRRLRR**	5	4795	**HHSTPHP**	3
545	**ATLPFVTDRQGW**	2	3741	**TPHQHDF**	9
421	**RIRDPRILLLHF**	6	1979	**TRILTIV**	8
389	**SFILFIRRLGRL**	12	1963	**HANLHHT**	4
332	**YRRLLIGMRRGG**	1	1923	**SPYPIRT**	4
303	**RRICRFIRICRVR**	1	1772	**HSPHPAD**	10
201	**RIRDPRILLLHF**	6	1521	**LLFIRRG**	6
189	**ASWQALALYAAGW**	1	1212	**IRGRIIR**	51

Data obtained from deep sequencing was analyzed at the DNA level; unique sequences were counted and divided into 7aa or 12aa categories according to origin of peptide libraries. The table shows the most relevant peptide sequences in terms of enrichment and functional activity determined at later stages. #Reads stands for the number of times a certain DNA sequence coding for a peptide appeared in the deep sequencing data and corresponds to the enrichment level. #Repeats stands for the number of different DNA sequences coding for the same peptide representing independently isolated phage clones. Colored amino acids represent different peptide motifs significantly enriched in both the 7aa and 12aa libraries and therefore selected by at least two independent experiments.

### Bioinformatic peptide motif analysis

Next, we performed a more comprehensive bioinformatics analysis to identify consensus motifs.

The 12aa library contains longer peptides and possibly more specific interactions between peptide and protein, but represents only a fraction of the possible library complexity (20^12^), whereas the shorter 7aa contains almost all possible peptides. Notably, comparison of sequences obtained from the two libraries revealed several common motifs shared by both libraries, supporting the validity of those motifs.

To identify enriched peptide sequence motifs, an algorithm was developed that checks the amino acid sequence in a growing window of peptide length. This algorithm scores each peptide, integrating the number of different nucleotide sequences that translate into the same peptide with the occurrences of each such sequence. Furthermore, it clusters the peptides by scoring the sequence similarity between different peptides, identifies groups of related peptide sequences, and extracts a consensus.

Candidate peptides were defined as those with occurrences of ≥ 0.2%: this resulted in 78 peptide motifs, which could be clustered into 40 groups by their blastp similarities and occurrence of a short similar motif. Figure 1E shows the top 8 scoring motifs.

### Functional screening of peptides

**Figure 2:**
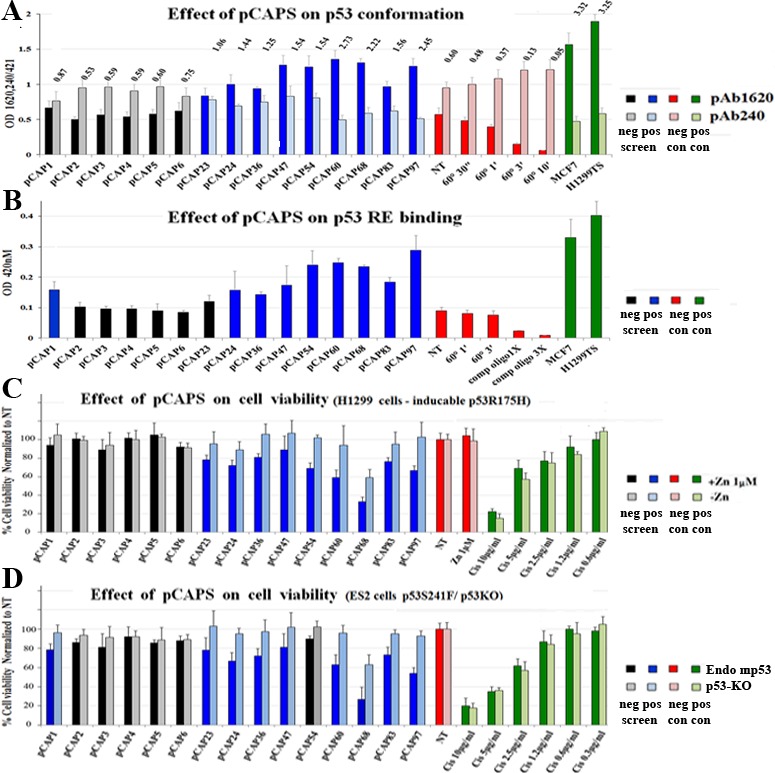
Screening for functional peptides **A.** Bar graph demonstrating representative ELISA experiments for determining the effect of selected peptides on the conformation of mutp53 in H1299-p53^R175H^ cell extract, as determined by immunoassay. Cell extracts were added to ELISA plates coated with the indicated antibodies to allow mutp53 to react with the peptides and antibodies. αp53-HRP Ab was used for assessment of p53 levels. Numbers represent ratio of absorbance between the PAb1620 and PAb240 samples. All reads were normalized to the control PAb241 reading of each extract. MCF7 (WTp53) and H1299-mutp53^A135V^ (tsp53) cells were used as positive controls for the WTp53 conformation (1620/240 ratio equals or exceeds 5:1). **B.** - Bar graph demonstrating representative ELISA experiments of determining the effect of selected peptides on the DNA binding activity of mutp53 in H1299-p53^R175H^ cell extract. 96 well plates were coated with anti-p53 antibody, cell extracts containing p53 were reacted with oligonucleotides that contain a p53RE consensus binding site, labeled with biotin, in the presence or absence (NT) of test peptides. Streptavidin-HRP is used to quantify the amount of oligos in the well. TMB assay was performed to determine bound mutp53 levels (450nm). MCF7 and the H1299-mutp53^A135V^(TS) cells serve as positive controls for WTp53. **C.**and **D.** - Bar graphs illustrating the effect of various selected peptides on mut-p53 dependent expression on cell viability. C represents H1299 p53-null (light blue bars) and H1299 stably overexpressing mutp53^R175H^ (blue bars). D, ES2 cells expressing endogenous mutp53 (blue bars) and ES2 cells knocked out for p53 by CRISPR construct (light blue bars). Cell lines were treated with selected peptides, Cis-platinum was used as positive control for cell death. 48 hours after treatment, cells were washed with PBS, and the remaining attached cells were stained with Crystal violet and washed 4 times with PBS. Stained cells were dissolved in 10% acetic acid and plates were taken for optical density measurement at 595nM.

A total of close to 350 peptides, deduced from the phage sequences, were synthesized and subjected to several alternative complementary methods of semi high-throughput functional screening; we refer to these peptides as pCAPs (p53 conformation activating peptides). First, we used cell-free ELISA assays to evaluate the effect of individual peptides on mutp53 conformation and DNA binding *in vitro*. For conformation analysis (Figure 2A), microtiter plates were coated with PAb240, PAb1620 or PAb421 as control, then the effect of each peptide on reactivity of mutp53 with these antibodies was examined; WTp53 served as a positive control for PAb1620 reactivity.

Figure 2A illustrates a representative analysis performed with an extract of H1299 cells stably overexpressing mutp53^R175H^. Extracts were prepared at a concentration of 750ng/μl and then reacted for 2 hours with different peptides. Plates coated with the different antibodies were then washed and blocked, and extracts pre-incubated either with or without peptides were added for two additional hours. After removal of extracts, plates were washed and incubated with HRP-conjugated anti-p53 antibody. Finally, HRP activity was quantified by optical density at 450nm. MCF7 cells (expressing WTp53) and H1299-tsp53^A135V^ cells, stably expressing a temperature sensitive p53 mutant that has mutant conformation at 37°C but WT conformation at 32°C [31], were used as positive controls for the WTp53 conformation (1620/240 ratio 5:1). Of note, although H1299-p53^R175H^ extracts react preferentially with PAb240, they still maintain some PAb1620 reactivity (1620/240 ratio 1:2). To determine whether this represents non-specific background binding or actual residual WT folding, we heat-denatured the extracts by incubation at 60°C for increasing periods. As seen, such treatment caused a gradual increase in PAb240 reactivity and decrease in PAb1620 reactivity, indicating that the p53^R175H^ in these cells is partly folded in a WT conformation under these experimental conditions. Peptides that stabilize mutp53 in a WT conformation are expected to increase binding to PAb1620 while decreasing PAb240 binding. Importantly, incubation with several of the tested peptides, including pCAPs 47, 54, 60, 68 and 97, increased the reactivity of p53^R175H^ with PAb1620 and decreased its reactivity with PAb240. Thus, some of our peptides can indeed stabilize mutp53 in a WT-like conformation.

Next, we assessed the ability of our selected peptides to restore sequence-specific DNA binding to mutp53. To that end we employed an ELISA kit (R&D) in which cell extracts containing p53 are reacted with a biotinylated p53RE oligonucleotide and then incubated in wells coated with anti-p53 antibody, which is expected to trap the p53 protein and its associated p53RE DNA. The amount of p53RE retained in each well *via* p53 binding is quantified by incubation with streptavidin-HRP. Peptides that restore the DNA binding activity of mutp53 are expected to significantly enhance the signal. A representative experiment is shown in Figure 2B. Again, extracts of MCF7 and H1299-tsp53^A135V^ cells served as positive controls for WTp53 DNA binding activity. As seen, H1299-p53^R175H^ extracts exhibited some background binding, which was further reduced by incubation with competing non-labeled oligonucleotide. Reassuringly, several peptides (e.g. pCAPs 54, 60, 68 and 97) elicited a marked increase in the binding of mutp53 to the p53RE, approaching the signal intensity obtained with MCF7 cells. Importantly, there was substantial overlap between the peptides that stabilized the WT conformation (Figure 2A) and those that increased specific DNA binding (Figure 2B), further reinforcing the conclusion that those peptides stabilize mutp53 in a functional state. This was not seen with the randomly selected peptides 1-6 (black bars).

It was particularly important to determine whether the mutp53 reactivating effects of the selected peptides could be exerted also within live cells. In view of the relatively large number of peptides that had to be tested individually, we employed a crystal violet-based viability assay. This simple assay measures the total number of cells, thus reflecting the combination of both cell death and growth arrest effects. H1299 cells transfected with p53^R175H^ under the control of a zinc-inducible promoter (Figure S1E) were plated in 96-well plates. Half of the wells were treated with Zn^++^ (1μM) to induce p53^R175H^ expression, and peptides were added 6 hours later; treatment with different concentrations of cisplatin served as a positive control. After 48 hours of treatment cells were stained with crystal violet and OD at 595nm was determined.

A representative experiment is shown in Figure 2C. Values on the Y-axis reflect the number of cells in the treated wells, normalized to the non-treated samples (NT; red bars). The cytotoxic effect of cisplatin was easily visible at the higher drug concentrations (green bars). Notably, several peptides had a clear inhibitory effect on the mutp53-expressing cells (dark blue), while affecting less, or not at all, the p53-null cells (light blue). Peptides 1-6 did not reveal such pattern, attesting to the specificity of the effect.

We also performed a similar analysis on ES2 ovarian cancer cells, which express endogenous mp53^S241F^, compared to ES2 cells in which p53 was stably knocked out using CRISPR/Cas9 (ES2 p53KO Figure S1F), to control for specificity for mutp53 (Figure 2D). In ES2 cells, too, some of the peptides caused a significant reduction in cell numbers, more prominent in the mutp53 expressing cells than in their p53KO counterparts. Importantly, although the H1299 and ES2 models are very different, the same peptides (e.g. pCAPs 24, 36, 54, 60, 83, 97) tended to exert a mutp53-specific effect in both. Overall, we performed this analysis on several different mutp53-expressing human cancer cell lines, and the results are summarized in Table S3. Based on this comprehensive picture, we reduced the number of most promising candidate lead peptides to 30. These peptides were then synthesized with an 8 arginine C-terminal tail for enhanced delivery across cell membranes (pCAPs 130-160); for clarity, peptides are referred to by their original number, with an R for poly arginine (see Table S3).

### Active peptides share similarity with p53-interacting proteins

We next asked whether any of the peptides that reactivate mutp53 bear similarity to sequences of human proteins known to interact with p53; such similarity may provide further validation that the peptides selected under artificial *in vitro* conditions can indeed interact with p53 in a functionally relevant manner. Moreover, the protein structure and surrounding sequence might be helpful in the rational design of improved peptides. Using BLAST (Basic Local Alignment Search Tool), we introduced the peptide motifs as query sequences against a human protein sequences database. We identified over 30 different proteins containing regions with varying degrees of similarity to selected peptide motifs. Many of these proteins had been shown previously to physically interact with p53, while others were reported to be involved in the p53 signaling pathway, either upstream or downstream of p53. Several motifs were found to have a very high degree of homology to known p53 interactors; for example, pCAP-97 (WNHHHSTPHPDH) and pCAP-250 (myr-RRHSTPHPD) (see later) have 100% homology to RAD9 (p-value of 10^−8^), shown to interact with and activate p53 [45]. Based on this information, and guided by the primary sequence and the three dimensional crystallographic data of the corresponding p53-interacting proteins, we then produced an additional set of peptides that resemble more closely the relevant regions of those proteins (pCAPs 200-326). N-terminal myristic acid and 2-4 arginine residues were also added to some peptides to increase their intracellular delivery. These peptides were subjected to functional assays as before, and the results are included in Tables 2 and S3.

**Table 2:**
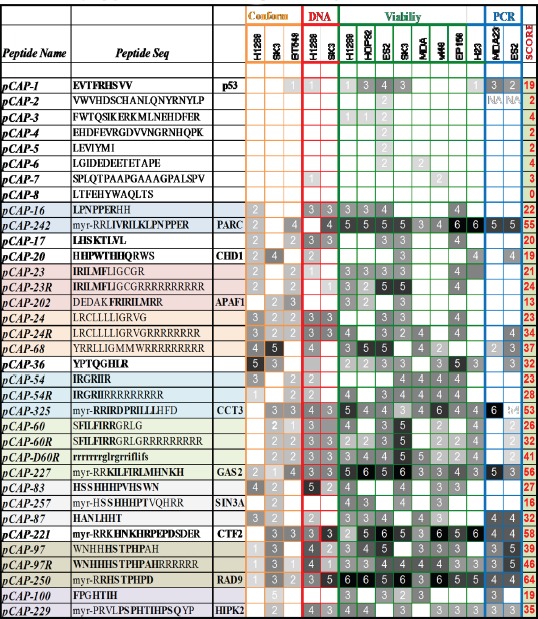
Summary of peptide functional screens and sequence similarity

Results of a variety of functional assays performed on representative synthesized peptides, presented as a heat map. Peptides were evaluated for their effect in each assay and assigned a score and corresponding color according to performance compared to positive control (score 0- no effect-white – score 6- as or more effective than positive control- black). Assays included mutp53 conformation (orange), mutp53 DNA binding (red), viability of mutp53 expressing cells and induction of p53 target genes by qRT-PCR (blue). Peptide sequences are aligned and have a background color according to similarity to known p53 interacting proteins and to each other. Since we changed peptide sequences along the project, in an attempt to yield more effective sequences according to performance in the different assays, table 2 also reflects this “evolutionary” process. The first set of peptides (pCAPs 1-160) corresponded to sequences identified by phage display. Then, lead sequences were modified to resemble p53 interacting proteins. Additionally, Arginines and N-terminal myristoil were added to enhance delivery, giving rise to pCAPs 200-326. Highlighted amino acids are similar to motifs found in p53 interacting proteins.

### Effect of peptides on mutp53 transcriptional activity

**Figure 3:**
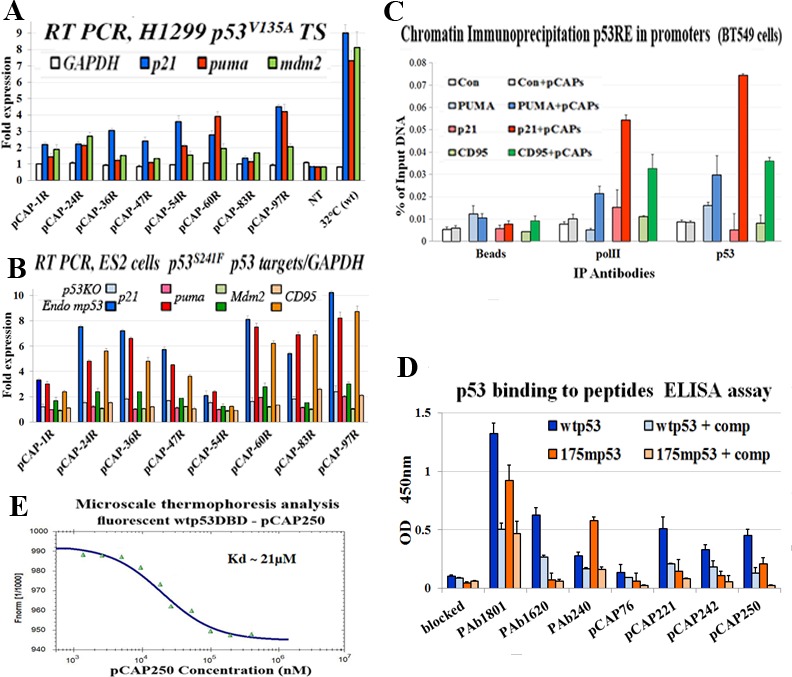
Peptides binding to p53 and their effect on the expression of p53 target genes **A.** - Bar graph illustrating the effect of selected peptides on activation of mutp53 by measuring transactivation of p53 target genes as determined by qRT-PCR. H1299 cells stably transfected with mutp53 (ts) A135V were used. The indicated peptides were added directly to the medium at a concentration of 5ug/ml and cells were then either moved to 32°C or returned to 37°C. 18 hours later cells were harvested, extracted for RNA, cDNA was synthesis subjected to qrt time PCR analysis. The expression level of 3 representative p53 target genes, p21, PUMA and Mdm2, were examined. The figure illustrates the relative fold induction of transcription of the cells treated with the selected peptides as compared to non-treated cells. **B.** - Bar graph illustrating the effect of selected peptides on activation of mutp53 by measuring transactivation of p53 target genes as determined by qRT-PCR. ES2and ES2-p53KO cells were treated with indicated peptides 5ug/ml, for 18 hours. Cells were harvested, and cDNA was subjected to qRT-PCR analysis. Expression of 4 representative p53 target genes, p21, PUMA, Mdm2 and CD95, was examined. The figure illustrates the relative fold induction of transcription in cells treated with the selected peptides compared to non-treated cells. **C.** - Binding of mutp53 to promoters of representative p53 target genes in live cells, assessed by chromatin immunoprecipitation. BT-549 breast cancer cells endogenously expressing mutant p53^R249S^ were treated for 5 hours with a mix of 3 pCAPs - 250, 242 and 325. Cells treated with a mix of inert peptides served as a negative control. DNA cross-linked p53 was immunoprecipitated, and binding to the p53 responsive elements of the *PUMA*, p21, *CD95* and *MDM2* gene promoters was quantified by qPCR. Results were normalized to input total DNA. Cell extracts immunoprecipitated with beads without antibody (beads) served as negative controls. A genomic segment containing no p53RE served as negative control (white and gray bars). **D.** - ELISA analysis of the binding of WTp53 and mutp53 to lead peptides. Peptides were conjugated to the bottoms of the wells of 96 well plates, employing a commercial conjugation kit (TAKARA). Control wells were coated with the αp53 monoclonal antibodies PAb1801, PAb1620 and PAb240. Recombinant WTp53 or mutp53R175H was added to the wells and incubated with the bound peptides or antibodies. Where indicated, soluble peptides were added as competitors (+ comp), to confirm the specificity of the binding to p53. pCAP-76 served as a negative control peptide. After removal of recombinant protein, plates were washed and incubated with αp53-HRP followed by TMB and optical density determination. Results are presented as relative absorbance at 450nm. **E.** - Microscale thermophoresis (MST) analysis of the binding of fluorescently labeled WTp53 DBD and pCAP-250. See Materials and Methods for details.

Since p53 works primarily as a transcription factor, we tested the ability of lead peptides to induce p53 target gene activation. To that end we used H1299-tsp53^V135A^ cells, which express temperature sensitive (ts) p53 and allow examination of WT and mutant p53 states in the same cellular background. In the representative experiment shown in Figure 3A, cells were exposed to the indicated peptides (5μg/ml) and then either transferred to 32°C or placed back at 37°C. Cells were harvested 18 hours later, and mRNA was extracted and subjected to qRT-PCR analysis. Expression levels of 3 representative p53 target genes, p21, *PUMA* and *Mdm2*, were examined. Values were normalized to non-treated cells at 37°C, and expression at 32°C, where the ts-p53 is in a WT conformation, served as positive control. As expected, temperature shift to 32°C greatly increased expression of the 3 target genes. A similar analysis was performed on ES2 ovarian cancer cells, expressing endogenous mp53^S241F^, compared to ES2 p53KO cells (Figure 3B).

Remarkably, several candidate lead peptides (pCAP-24R, pCAP-54, pCAP-60R, pCAP-97R) elicited a significant increase in the expression of the p53 target genes in both cellular models (Figure 3A, 3B), indicating that they can indeed restore not only WTp53 conformation but also WTp53 transcriptional activity within cells.

### Lead peptides induce binding of mutp53 to promoters of p53 target genes in live cells

Next, we performed chromatin immunoprecipitation (ChIP) analysis to examine the ability of the peptides to restore the binding of mutp53 to p53REs within live cells. Breast carcinoma BT-549 cells, endogenously expressing mutant p53^S249R^, were treated for 5 hours with a mix of pCAP-250, pCAP-308 and pCAP-325. Identical cultures were treated in parallel with a mix of control peptides (Figure 3C, light-colored bars). All cultures were then subjected to ChIP with antibodies against RNA pol II and p53; as negative control, extracts were immunoprecipitated with beads only (Beads). The precipitated DNA was subjected to qPCR analysis with primers corresponding to specific p53REs. As seen in Figure 3C, p53 binding to cognate responsive elements in the *PUMA*, p21 and *CD95* genes was elevated 2.34, 9.78 and 4.54 fold, respectively, compared to control peptides, following pCAP treatment. Enhanced p53 binding was associated with recruitment of Pol II to the same genomic region. Hence, pCAPs enable mutp53 to bind p53REs within cells, recruit Pol II and drive transcription of WTp53 target genes.

### Binding of lead peptides to p53

In theory, one might envisage two different mechanisms for reactivation of mutant p53 by our peptides. Peptides may bind preferentially to the misfolded mutant p53, forcing it to adopt a more properly folded state resembling WTp53. Alternatively, the peptides may actually bind preferentially to p53 when it is in the properly folded state resembling WTp53. Assuming that there is a dynamic equilibrium between the folded and misfolded states of mutp53 (see Figure 2A), the peptide may stabilize the small fraction of properly folded molecules and prevent them from becoming again misfolded, gradually shifting the population equilibrium towards a WTp53 conformation. To distinguish between these possible mechanisms, we conducted a direct binding assay in order to determine whether our lead peptides bound more efficiently to WT or mutant p53. To that end, an ELISA assay was performed, where peptides were conjugated to plastic wells, followed by incubation with either recombinant WTp53 or mutp53 and then the assay was developed with HRP-conjugated anti-p53 antibody. Wells coated with different anti-p53 monoclonal antibodies served as internal controls. As expected, mutp53 bound preferentially to PAb240, whereas WTp53 bound preferentially to PAb1620 (Figure 3D). Importantly, all three tested lead peptides - pCAP 221, 242 and 250- recruited preferentially WTp53, while the recruitment of mutp53 was 50%-80% lower. This argues strongly that these peptides prefer the WT conformation of p53, supporting the second possibility above, namely that they bind to the properly folded WT-like (rare) conformation of mutp53 and stabilize the population gradually in that conformation.

Although the ELISA analysis in Figure 3D was performed with full length p53 proteins, it was highly plausible that they actually bind within the p53 DBD and stabilize its WT conformation. To confirm this prediction and assess the strength of the interaction between lead peptides and the correctly folded DBD, we employed microscale thermophoresis (MST) analysis, which is based on the altered movement of a protein in a temperature gradient when bound to other molecules. Figure 3E illustrates a representative experiment monitoring the binding of fluorescently labeled WTp53 DBD and pCAP-250, showing clearly concentration-dependent direct binding of pCAP-250 to the WTp53 DBD, with a Kd of ∼ 21μM (average of 3 experiments).

### Peptides trigger apoptosis in correlation to activation of WTp53 target genes

**Figure 4:**
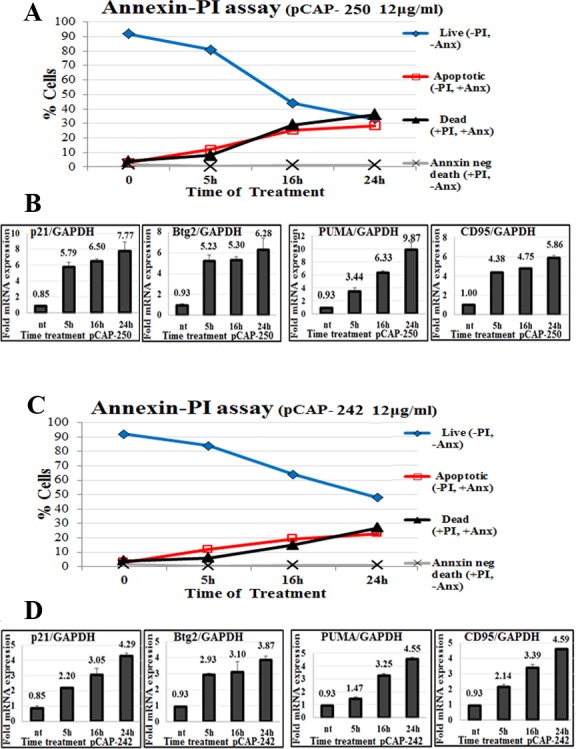
Peptides trigger apoptosis in correlation to activation of WTp53 target genes **A.**, **C.** - Apoptosis of ES2 cells treated with peptides. At the indicated time points, peptides were added directly to the medium of growing ES2 cells at a concentration of 12ug/ml. Cells were harvested and stained with Annexin FITC to detect apoptotic cells, and PI (propidium iodide) staining for dead cells. Stained cells were then analyzed by flow cytometry. A total of 10,000 cells were counted for each sample. **B.**, **D.** - Expression of p53 target genes p21, *PUMA*, *BTG2* and *CD95* in ES2 cells following peptide treatment. Either pCAP-242 (figure 4D) or pCAP-250 (Figure 4B) was added directly to the medium at a concentration of 12μg/ml. At the indicated time points cells were harvested and RNA subjected to qRT-PCR analysis.

To assess whether pCAPs can trigger apoptosis in mutp53 expressing cells, we subjected non-fixed ES2 cells to Annexin V staining in conjunction with propidium iodide (PI). In such analysis, cells negative for both PI and Annexin V (−PI, −Annexin) are considered live, cells negative for PI and positive for Annexin V (−PI, +Annexin) are going through early stages of apoptosis, cells positive for PI and Annexin V (+PI, +Annexin) are considered already dead by apoptosis, and cells positive for PI and negative for Annexin V (+PI, −Annexin) are assumed to have undergone non-apoptotic death. As seen in Figure 4A and 4C, pCAP-250, and to a somewhat lesser extent pCAP-242, elicited a rapid increase in apoptotic cells followed by cell death, along with significant transactivation of WTp53 target genes (Figures 4B, 4D).

### Lead peptides exert anti-tumoral effects *in vivo*

To determine whether the observed mutp53-reactivating effects of our lead peptides can be translated into anti-tumoral activities *in vivo*, we subjected several subcutaneous human xenograft models in nude mice to intratumoral injection of active or control peptides. By using tumor cells stably expressing luciferase, we were able to monitor effects on tumor growth by IVIS live imaging. For each xenograft model, we first tested the effect of the lead pCAPs on the pertinent cell line in culture (Figure S2); the most effective peptides were then selected for *in vivo* analysis, either as single peptides or as a mix of three peptides.

**Figure 5:**
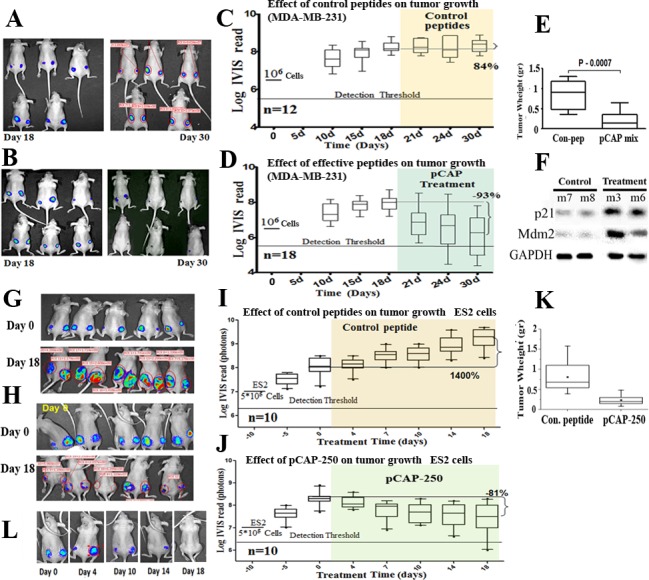
Effect of lead peptides on tumor growth *in vivo*; breast and ovarian cancer models **A.**-**D.** - Effect of indicated peptides in a mouse breast cancer xenograft model.10^6^ MDA-MB-231 breast cancer cells, expressing endogenous mutp53 and stably expressing luciferase, were injected into the hips of nude mice. When tumors reached visible size, mice were treated by intra-tumoral injection, three times a week, with either a mixture of 3 control peptides that showed no phenotype *in vitro* (pCAPs 76, 77 and 12; 2μg of each peptide) or a mixture of 3 test peptides that exhibited mutp53-reactivating ability (pCAPs D60R, 24R and 174; 2μg of each peptide). **A.** - Live imaging of control group mice at the beginning of treatment (day 18) and at termination of the experiment (day 30). **B.** - Live imaging of mice treated with effective peptide mix at the beginning of treatment (day 18) and at termination of the experiment (day 30). **C.** - (control group mice) and **D.** (effective pCAP mix group): Logarithmic scale box-plot showing the luciferase readings in tumors as a function of time; average (horizontal line), standard deviation (box), highest and lowest reads (error bars) are shown, before (until day 18) and after initiation of treatment. The background threshold detection level of the IVIS system in this experiment was about 5×10^6^ photons. **E.** - Box-plot of excised tumor weights at termination of the experiment. Average weight (horizontal line), standard deviation (box), highest and lowest reads are shown.**F.** - Western blot analysis of two p53 target gene products, p21 and MDM2. Part of the excised tumors of mice #7 and #8 treated with control peptides and mouse #3 treated with effective peptide mix were homogenized and lysed. Protein concentration was determined using Bio-Rad reagent. 50μg protein of each sample was loaded and subjected to Western blot analysis with antibodies against p21 and MDM2. **G.**-**K.** - *In-vivo* effect of indicated peptides in a mouse xenograft model. 5*10^5^ ES2 cells expressing luciferase were injected into the hips of nude mice. Bioluminescence was measured. 10 days after injection, mice were randomly divided to 2 groups and injected intratumorally, three times a week, with either a mixture of 2 control peptides (pCAPs 76 and 12; 5μg of each peptide) or pCAP-250 (10μg). **G.**, **H.** - Live imaging of control group mice and pCAP-250 treated mice, respectively, at the beginning of treatment (day 0) and at termination of experiment (day 18). **I.** (control mice) and **J.** (effective pCAP-250 group): box-plot showing the luciferase readings in tumors as a function of time; average (horizontal line), standard deviation (box), highest and lowest reads are shown, before (until day 0) and after initiation of treatment. The background threshold detection level of the IVIS system was about 5×10^6^ photons. **K.** - Box-plot of excised tumor weights at termination of the experiment. Average weight (horizontal line), standard deviation (box), highest and lowest reads are shown.**L.** - Live imaging of a single mouse treated with pCAP-250 over the indicated time points.

The first *in vivo* model consisted of MDA-MB-231 breast cancer cells, endogenously expressing p53^R280K^. A total of 15 mice were injected subcutaneously, in both hips, with 1×10^6^ cells. Treatment was administered 18 days post-injection, when tumors reached visible size. Control treatment (6 mice) was composed of a mixture of 3 control peptides (pCAPs 76, 77 and 12). Mice of the treatment group (9 mice) were injected with a mixture of 3 active peptides (pCAPs 174, 155 and D60R). Peptides were injected intratumorally three times a week, 2μg per tumor for each peptide. As shown in Figure 5A-5D, the mixture of three p53 reactivating pCAPs, but not control pCAPs, caused a significant decrease in the number of tumor cells, deduced from luciferase intensity. Remarkably, as shown in Figure 5B, 12 days after beginning of the treatment (4 injections) the average tumor luminescence was decreased by 93%, with 11 out of 18 tumors showing a complete response. Furthermore, 6 mice showing a complete response were kept alive for an additional 60 days after cessation of the treatment and were monitored for tumor recurrence; no recurrence was detected. Only one of the 18 tumors injected with active pCAPs failed to show a measurable response, probably due to its relatively large size before beginning of treatment.

At the end of the experiment, tumors were excised, weighed and subjected to Western blot analysis. As seen in Figure 5E, tumors in the treatment group were substantially smaller. Notably, when compared to control peptide-treated tumors, tumors treated with activating pCAPs displayed markedly higher levels of the p53 targets p21 and MDM2 (Figure 5F). This strongly indicates activation of mutp53 *in vivo* following pCAP treatment.

We performed two additional preclinical experiments with breast cancer models. The first experiment, again with MDA-MB-231 cells, is depicted in Figure S3A-S3D. The second experiment was done with SKBR3 cells (Figure S3E-S3H). In this experiment, treated tumors showed a 50% reduction within 24 days.

A similar analysis was performed with ES2 ovarian cancer cells. The experiment was conducted on 10 mice, each injected subcutaneously with 5×10^5^ cells. After 10 days, when tumors reached visible size, the mice were randomly divided into 2 groups: a control group, treated with a mix of 2 control pCAPs, and a group treated singly with pCAP-250. The results are shown in Figure 5G-5L. Figure 5I, 5J shows tumor growth over time of the control and pCAP-250 groups as measured by live imaging. As seen, after peptide administration (10μg peptide/tumor, 3 injections per week), the control tumors showed an average 14 fold increase in size. In contrast, tumors in the pCAP-250 treatment group showed an 81% size decrease, and one of five mice showed a complete response (Figure 5L). This mouse was kept alive for 30 days after cessation of treatment, without recurrence of tumors. The decrease in tumor size is reflected also by the weight of the excised tumors, and is statistically significant (*p* = 0.002, Figure 5K).

**Figure 6:**
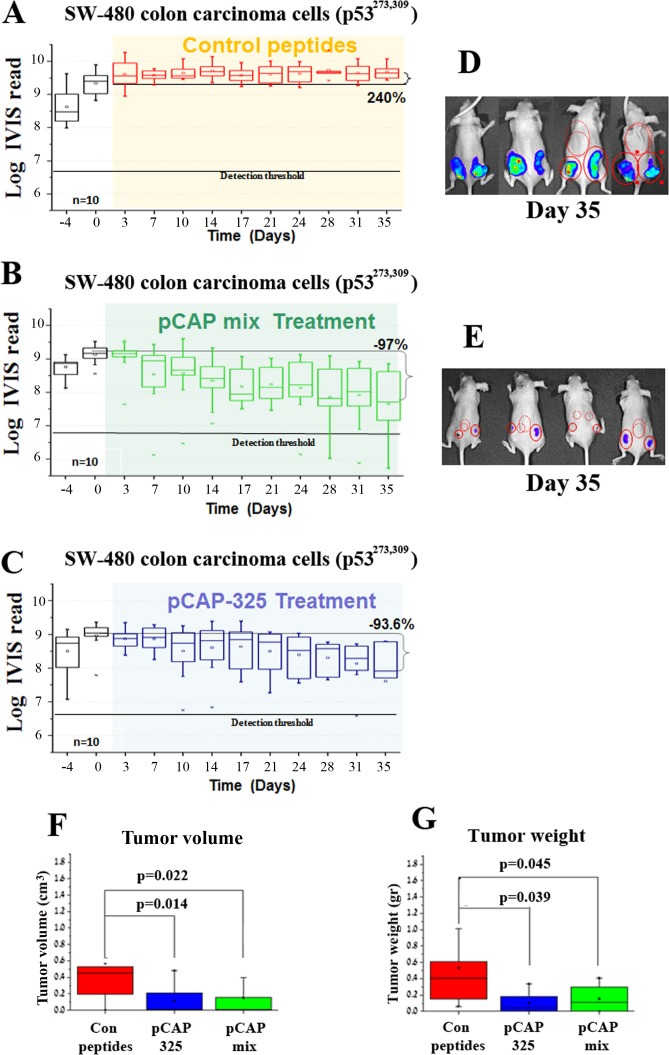
Effect of lead peptides on tumor progression *in vivo*; colon cancer xenograft model *In vivo* effect of the indicated peptides in a mouse xenograft model of SW-480 colon cancer cells. 10^6^ cells were injected and tumors were allowed to establish for 10 days. Mice were then randomly divided into 3 groups and injected intratumorally, three times a week, with either a mixture of 3 control peptides (pCAPs 76, 77 and 12; 2μg of each peptide), a mixture of 3 test peptides that exhibited mutp53-reactivating ability (pCAPs 250, 308 and 325; 2μg of each peptide) or pCAP-325 (6μg). **A.**-**C.** - Logarithmic scale graph demonstrating the average luciferase readings in tumors as a function of time, before and after initiation of treatment (colored background). **D.**, **E.** - Box plot of tumor volume and weight, respectively.

The lead peptides were evaluated also in a colon carcinoma xenograft model, employing SW480 cells that harbor p53^R273H, P309S^. The experiment was conducted on 15 mice, randomly divided into 3 groups: a control group treated with a cocktail of 3 ineffective peptides, a group treated with a cocktail of 3 effective pCAPs (250, 308, 325) and a group treated singly with pCAP-325. Tumors were allowed to reach visible size for 10 days before onset of treatment. Figure 6 shows tumor growth over time as measured by live imaging; the first day of treatment is marked as day 0. As seen, while the control tumors showed an average increase of 2.75 fold (Figure 6A), tumors in the mix treatment group showed a 96.7% size decrease (Figure 6B). Similarly, tumors in the pCAP-325 group showed an average decrease of 93.6% (Figure 6C). Figures 6F, 6G show the mean weight and volume of excised tumors.

Overall, 5 pre-clinical experiments were performed. In all those experiments, mice treated with lead peptides showed a significant decrease in tumor size, while tumors treated with control peptides continued to grow (Table 3).

**Table 3:** Summary of performed pre-clinical experiments

Experiment number	1		2		3		4			5	
Cell line type	**MDA-MB-231 p53^R280H^**		**MDA-MB-231 p53^R280H^**		**SKBR3 p53^R175H^**		**SW-480 p53^R273H,P309S^**			**ES2 p53^R241H^**	
Group	Treatment pCAPs	Control pCAPs	pCAPs 250, 60R, 154	Control pCAPs	Treatment pCAPs	Control pCAPs	pCAP 325	pCAPs 250, 325, 317	Control pCAPs	pCAP 250	Control pCAPs
Number of samples	10	6	18	12	10	10	10	10	10	10	10
**IVIS average ratio to day 0**	**22.0%**	**321.0%**	**6.9%**	**184.0%**	**43.7%**	**1000.0%**	**6.4%**	**3.3%**	**275.4%**	**19.0%**	**1400.0%**
% of tumors total regression	40%	0	55%	0	0	0	20%	40%	0	20%	0
Samples average size	0.27	1.26	0.13	0.89	0.15	0.38	0.12	0.15	0.87	0.15	0.48
Samples average weight	0.29	1.11	0.15	0.94	0.24	0.53	0.1	0.15	0.53	0.21	0.53

## DISCUSSION

Protein folding in general, and of p53 in particular, depends on many parameters; temperature, ionic strength, amino acid composition and interaction with other proteins or peptides. For simple protein domains, a two state equilibrium exists between folded and unfolded conformations, whereas multi-domain proteins like p53 often exhibit intermediate states. However, the process of folding and unfolding is reversible [46]. Since p53 is intrinsically unstable, most amino acid substitutions in the p53 DBD share a common effect of shifting the equilibrium towards an unfolded intermediate state under physiological conditions. This equilibrium can be regulated by molecular chaperones, as exemplified in embryonic stem cells harboring endogenous mutant p53, yet mostly expressing the wild type conformation of the protein [29]. We propose that the destabilizing effects of point mutations can be reversible through interaction with peptides that bind to p53, thermodynamically stabilize the correct p53 protein folding, and hence restore tumor suppressor function.

We describe an innovative approach for the identification of mutp53 reactivating peptides through functional screening of phage display libraries. The competitive nature of phage display allows rapid screening of high complexity peptide libraries without examination of each individual peptide. In our protocol, the use of alternating selection methods greatly reduces the number of non-specific false positives, while deep sequencing enables recovery of the entire peptide repertoire and allows a much more comprehensive analysis of consensus motifs. Interestingly, the efficacy of individual pCAPs was cell type-dependent (figure S2); this might indicate that different peptides might have preferential impact on different p53 mutations, although it is plausible that differences in the cell context also play a role. Evaluation of the efficacy of different peptides on an isogenic cellular background using different p53 mutants is required in order to resolve this issue. Nevertheless, several peptides caused significant mutp53-dependent cell death in the majority of tested cell lines. Notably sequence alignment of lead peptides indicated that many of them bear homology to proteins known to interact with p53.

The mode of action of the various pCAPs still remains to be fully determined, and it is plausible that different groups of peptides may restore WTp53 activity by non-identical molecular mechanisms. Nevertheless, at least for some lead peptides such as pCAP-250, the presently available data strongly suggest that rather than binding to the misfolded mutant p53 protein and somehow forcing it back into the proper conformational state, the peptides actually interact preferentially with the WT p53 conformation (Figure 3). In a typical population of conformational mutant p53, the vast majority of molecules at any given time are probably in the misfolded state, whereas only a minority are properly folded. Yet, we propose that there exists a constant dynamic equilibrium between those states. By binding preferentially to mutp53 when it transiently adopts a WT conformation, our peptides might stabilize this conformation and gradually shift the population equilibrium towards it.

Despite major efforts to develop p53-reactivating compounds, there has been only limited success so far. The major factors that have hindered the clinical application of p53-based therapies are safety issues, low specificity to p53 resulting in relatively high toxicity, and insufficient phenotypic effect on mutp53. A therapeutic approach based on small peptides holds several important advantages; due to their high complexity, peptide drugs are more specific than small molecules, usually non-immunogenic and relatively inexpensive to manufacture. Moreover, peptides are not excluded from cells by multiple drug resistance mechanisms [47, 48]. For those advantages, many peptide-based drugs are under pre-clinical and clinical phase development [49]. Remarkably, the lead peptides isolated in our study showed impressive activity in preclinical models, reducing tumor size substantially in several different experimental models, and in some cases even leading to complete eradication of the tumor and preventing relapse even after cessation of the peptide treatment.

There are still major challenges to overcome before clinical application of these peptides can be considered [50]. Susceptibility to proteolytic degradation is a known limitation of peptide-based therapies; C-terminal amidation and the use of D-amino acids are potential ways to overcome this limitation. Delivery is also a considerable challenge; to date; we only administered the peptides by intratumoral injections. One should also overcome rapid renal clearance from the blood, with a consequent expected short half-life of small peptides in the circulation [51]. Treatment using intratumoral injections of peptides can be applicable to only a small portion of patients. Therefore, there is need to develop peptide modifications that will extend the retention of the peptides in the circulation and allow their systemic administration. Tumor targeting and increased intracellular penetration are also key needs that can potentially be met by a variety of available approaches. Despite these challenges, we believe that the peptides described here carry great potential for the treatment of many patients with tumors harboring mutant p53.

## MATERIALS AND METHODS

### Phage display

Phage display librar is used were commercially available, generated by New England Biolabs (NEB). Phage display procedure was performed according to manufacturer instructions. Briefly; Prior to immunoprecipitatation, beads were blocked. Panning was performed by allowing interaction between phage libraries 10^10^ phage and 1μg of either wtp53 His-WT53, mutp53^R175H^ or DBD-mutp53^R249S^, for 2 hours. Phage-p53 complexes were then immunoprecipitated using different biological agents covalently attached to beads; Nickel beads for His-WT53-phage complexes, Biotin-p53RE-DNA-Streptavidin beads for WT53-phage complexes, PAb1620-beads and LTag-PAb419-beads for p53^R175H^-phage complexes. Immunoprecipitation was performed for 2 hours at room temperature. Agarose beads were washed 10 times with PBS 0.5% Tween. Phage were eluted using either HindIII, EcoRI (Figure S2A) digestion 30min 37C° for phage-wtp53-p53RE complexes, or through competition based elution with an access of DBD-wtp53 30min RT for other phage-p53 complexes. Eluted phage were tittered (Table S2) and amplified through infection of early-log *E. coli* and extracted using PEG precipitation. The second and third rounds of panning were carried out in a similar manner with the same amount of phage, however the amount of p53 was reduced to 500ng and 200ng in the second and third rounds respectively.

### Sandwich ELISA

96-well plates were coated using 3 different anti p53 monoclonal antibodies, PAb421, PAb240 and PAb1620. Wells were incubated overnight (ON) with 100μl Ab (5μg/ml) in room temp (RT). The liquid was discarded, and the wells were washed 3 times with PBS, 200 μl. Next, blocking with 5% BSA in PBS for 2 hours (RT) was performed, followed by 3 washes in PBS. Samples of mutant and WT p53 proteins (100 μl, 10 μg/ml), together with control peptides or with test peptides 1-326 (5μg /ml), were incubated for 1.5 hours, and then added to the wells for1 hour at RT. 4 washes with PBST were performed. Next, HRP conjugated αp53 antibody (10μg/ml HAF1355 (R&D)) were added to the wells and incubated at RT for 1 hour. Plates were washed 3 times in PBST and TMB substrate solution (50μl each well, Thermo, (Cat. No. ES001-1L-K)) was added and incubated at 37°C for 20 min. The reaction was stopped with 2M Sulfuric acid (50μl). The absorbance was measured at 450 nm with a spectrophotometer. Protein concentration was determined by dividing the absorb an each sample to by the absorbance of PAb421 samples.

### Microscale thermophoresis (MST) analysis

Microscale thermophoresis analysis (MST) for binding of fluorescently labeled WTp53DBD and pCAP-250 was performed according to the manufacturer's instructions (NanoTemper Technologies). 40μg (20 μM) of purified wtp53DBD was labeled with the provided fluorescent blue dye. The fluorescent signal was evaluated and the amount of protein was calibrated to 4ng of protein per sample. 10 serial dilutions of pCAP-250 (highest concentration = 0.4mM) were prepared in 50mM Hepes buffer pH 6.1 containing 0.05% Tween. 4ng of labeled protein was added to each peptide sample and incubated for 30 minutes. Samples were centrifuged for 10 minutes at 3500g and loaded to capillaries. MST analysis was performed using the monolith NT.115 instrument. Fluorescence was measured at 50% MST power and detected at 20, 40, 60 and 80 LED power.

### Bioinformatics analysis

Sequenced 7aa and 12aa libraries were processed with the aid of the Galaxy program [52, 53] keeping only regions coding for the random peptide sequences and merging identical sequences while keeping their counts. The resulting sequences were translated and identical peptides merged, keeping the number and counts of different genotypes, total number of occurrences and their percentage in the library. Peptides that occurred at a frequency of at least 0.2% in a library were clustered using the blastp program [54]. Similar peptides were transformed into block multiple alignments, using the percentage occurrences as sequence weights [55]. The resulting blocks were used to query the peptide-clustered sequence files, and the top results were again transformed into blocks in the same way.

### CRISPR p53 knockout

Plasmid #42230, containing a *TP53* exon 3 single guide RNA (sgRNA), was from Addgene. ES2 cells were transfected using jetPEI reagent (Polyplus) according to the manufacturer's protocol. After 48 hours, cells were seeded in a 96 well plate as single cell clones. Single cell clones were expanded and their p53 status was examined by Western blot analysis, using the DO-1 anti-p53 antibody.

sgRNA sequences: F: 5′-CACCGCCATTGTTCAATATCGTCCG-3′

R: 5′-AACCGGACGATATTGAACAATGG-3′

### Crystal violet viability assay

Cells were cultured in 96 wells plates with 2500-4000 cells/well. Serial dilutions of different peptides were added and the plates incubated for additional 48 h at 37°C. Then medium was removed and cell viability was determined by staining the cells with crystal violet (0.05%) in methanol/PBS (1:5, v/v), for 10 min, followed by 3 washes with PBS. 10% acetic acid was added to each well for 10 min. OD was determined at 595 nm.

### Deep sequencing

Prior to sequencing, a PCR reaction was performed with primers flanking the inserted libraries. The 8 bases of each primer we randomized and were incorporated as a mixture of all four bases (Supplementary Data). Randomization of first bases was introduced since the “Solexa” sequencing equipment is incapable of sequencing repetitive sequences for the first few cycles. The PCR reaction yielded DNA in the required quantity 5μg and length (about 120 bp).

### DNA binding assay

For these experiments, a commercial p53/DNA binding kit of “R&D” (Cat-DYC1355-5 Lot-1273366FA) was used, in accordance with manufacturer guide lines. Briefly, 96 well plates we coated with anti-p53 antibody overnight. Cell extracts containing p53 are reacted with an oligonucleotide that contains a p53 consensus binding site (provided in the kit), labeled with Biotin, in the presence or absence (NT) of test peptides. WT p53 is expected to bind this DNA binding site as well as to the antibody coating the test wells of the plate. Excess p53 and oligos were washed away and streptavidin-HRP was used to quantify the amount of oligos in the well, which is proportional to the DNA bound by p53. TMB assay was performed to determine HRP (ES001-1L-K) levels (450nm).

### Chromatin immunoprecipitation (ChIP) analysis

Chromatin immunoprecipitation was performed as described [56] employing protein A beads cross-linked by DMP to anti p53 polyclonal antibodies, or anti RNA PolII antibodies. Beads only served as a non-specific control. DNA samples were extracted using PCR clean-up mini-columns (Quigen). Real-time PCR was performed using SYBR Green as described above.

### Preclinical testing of peptides

Mice (6 weeks athymic nude) were injected subcutaneously with 2×10^5^-10^6^ cells into each femur. All cell lines employed in these experiments stably express a luciferase reporter gene to enable monitoring of tumor growth by live imaging. 4-18 days later, when tumors reached visible size, the mice were randomly divided into several groups: a control group, treated with either a single control peptide or a mix of 2-3 control pCAPs, and groups treated with effective peptides, either a single peptide or a mix of three peptides. Peptides were administered by intratumoral injection of 6-10μg peptide per tumor in 40μl PBS, three times a week. Tumor growth over time was measured by live imaging, using the IVIS2000 system. Exposure time was calibrated to 20 seconds. 16 images were taken over 8 minutes and peak luminescence values were taken for each tumor. Experiments were conducted until tumors reached maximal allowed size of 1cm^3^, at which time were sacrificed and tumors extracted, measured and weighed.

### RT-PCR

RNA was obtained using Macherey-Nagel NucleoSpin RNA II Kit on cells pellet according to the manufacturer's protocol. Aliquots of 0.4-1 μg were reverse transcribed using Bio-RT 2000 (Bio-Lab) and random hexamer primers. QRT-PCR was performed on an ABI 7300 instrument (Applied Biosystems) using SYBR Green FastMix ROX (Quanta). RT-PCR primers (All primers sequences are presented 5′ to 3′):

## SUPPLEMENTARY MATERIAL FIGURES AND TABLES


