# Supplementary material for: Cancer therapeutic approach based on conformational stabilization of mutant p53 protein by small peptides
Source: Oncotarget. 2016 Mar 2;7(11):11817–37. doi: 10.18632/oncotarget.7857 (PMC4914250; doi:10.18632/oncotarget.7857)
Supplement: Supplementary file 1 [file oncotarget-07-11817-s001.pdf]

Cancer therapeutic approach based on conformational stabilization of mutant p53 protein by small peptides

Supplemental Material

Supplemental materials and methods

Purification of recombinant full length (FL) proteins from Sf9 cells: mutant $p53^{R249S}$, mutant $p53^{R175H}$ and WT p53:

$2x10^7$ Sf9 cells in log-phase were grown in nine 175 cm^2 flasks and incubated overnight at 27°C. Baculoviruses containing a recombinant p53 were added into each flask, and incubated for 72 hrs. Cells were scraped from the flasks and centrifuged at 4°C. The cell pellet was washed twice with ice-cold isotonic buffer (10mM Na_2HPO_4, pH 7.2, 130 mM NaCl, 1 mM DTPA). Cells were suspended in 50ml of Buffer A (20 mM Tris-HCl, pH 8.0, 12% sucrose, 2 mM EGTA, 2 mM PMSF, 5 mM DTT) with 0.2% Triton X-100 by gentle inversion. Nuclei were pelleted by centrifugation at 5600g for 8 min, and then were lysed by adding 20 ml of Buffer B (20 mM Tris-HCl, pH 8.0, 12% sucrose, 2 mM EGTA, 2 mM PMSF, 10 mM DTT + protease inhibitors) with 0.5M NaCl followed by vigorous vortexing and incubation for 20 min on ice. The nuclear lysate was transferred to ultracentrifuge tubes and centrifuged at 100,000g for 60min at 4°C. The supernatant was removed and diluted with Buffer B to a final concentration 0.04 M of NaCl, then centrifuged at 20,000g for 5 min at 4°C. The nuclear lysate was loaded onto a 5ml Hitrap Q FF (Amersham Pharmacia) ion-exchange column, pre-washed with 50 ml of buffer A. Then, the column was washed with buffers containing higher salt concentrations to elute

the protein. The protein was further purified by gel-filtration chromatography using a preparative Superdex 75 column (Amersham Pharmacia Biotech), pre-equilibrated with 20 mM sodium citrate pH 6.1, 150 mM NaCl, 10 μM $ZnCl_2$, and 10 mM DTT. Fractions containing purified protein were pooled together. The fractions obtained after each purification step were analyzed by dot-blot for the presence of mutant p53 and subsequently by SDS-PAGE followed by Coomassie blue staining to check the purity of the fractions.

Phage Display

The phage display libraries used were purchased ffom New England Biolabs (NEB). One library consists of linear hepta-peptides (PhD-7) and the other library consists of linear dodeca-peptides (PhD-12) (CAT NOs.: PhD-7, E8100S and PhD-12, E8110S, accordingly). The randomized peptide sequences in both libraries are expressed at the N-terminus of the minor coat protein pIII, resulting in a valency of 5 copies of the displayed peptide per virion. Both libraries contain a short linker sequence between the displayed peptide and pIII. Phage display procedures were performed according to the manufacturer instructions. Briefly, phage libraries were titered using the supplied *E. coli* host strain ER2738 which is a robust F+ strain with a rapid growth rate and is suited for M13 propagation. The supplied stock phage concentration was estimated to be 10^{12} phage/ml. Panning was performed by allowing interaction between 10μl of phage libraries (10^{10} phage) and 5μg of either WT53 His-wtp53, mutp53^{R175H} or DBD-mutp53^{R249S}, for 2 hours in 50mM Tris, 1% BSA. Phage–p53 generated complexes were immunoprecipitated by using different biological agents covalently attached to beads.

These included nickel beads for His-WTp53-phage complexes, Biotin-p53RE-DNA-Streptavidin beads for WTp53-phage complexes, and PAb1620-beads and LTAg-PAb419-beads for $p53^{R175H}$–phage complexes. Prior to immunoprecipitation, beads were blocked with blocking buffer: 0.1 M NaHCO3 (pH 8.6), 5 mg/ml BSA, 0.02% NaN3. Immunoprecipitation was performed for 2 hours at room temperature. Agarose beads were washed 10 times with PBS+0.5% Tween. Phage were eluted using either HindIII, EcoRI digestion for 30 min at 37C° for phage-WTp53-p53RE complexes, or through competition-based elution with an excess (5µg) of DBD-WTp53 for 30min at RT for other phage-p53 complexes. Eluted phage were titered (Table S2) and amplified through infection of 20 ml early-log *E. coli* host strain ER2738 and incubated at 37°C with vigorous shaking for 4.5 hours. Phage were extracted using PEG precipitation (15 minutes at 10,000 rpm at 4°C) and resuspended in 200 µl TBS, 0.02% NaN3. The amplified eluate was titered according to the manufacturer's instructions on LB/IPTG/Xgal plates. The second and third rounds of panning were carried out in a similar manner, using the same amount of phage; however, the amount of p53 was reduced to 3µg and 1µg in the second and third rounds, respectively.

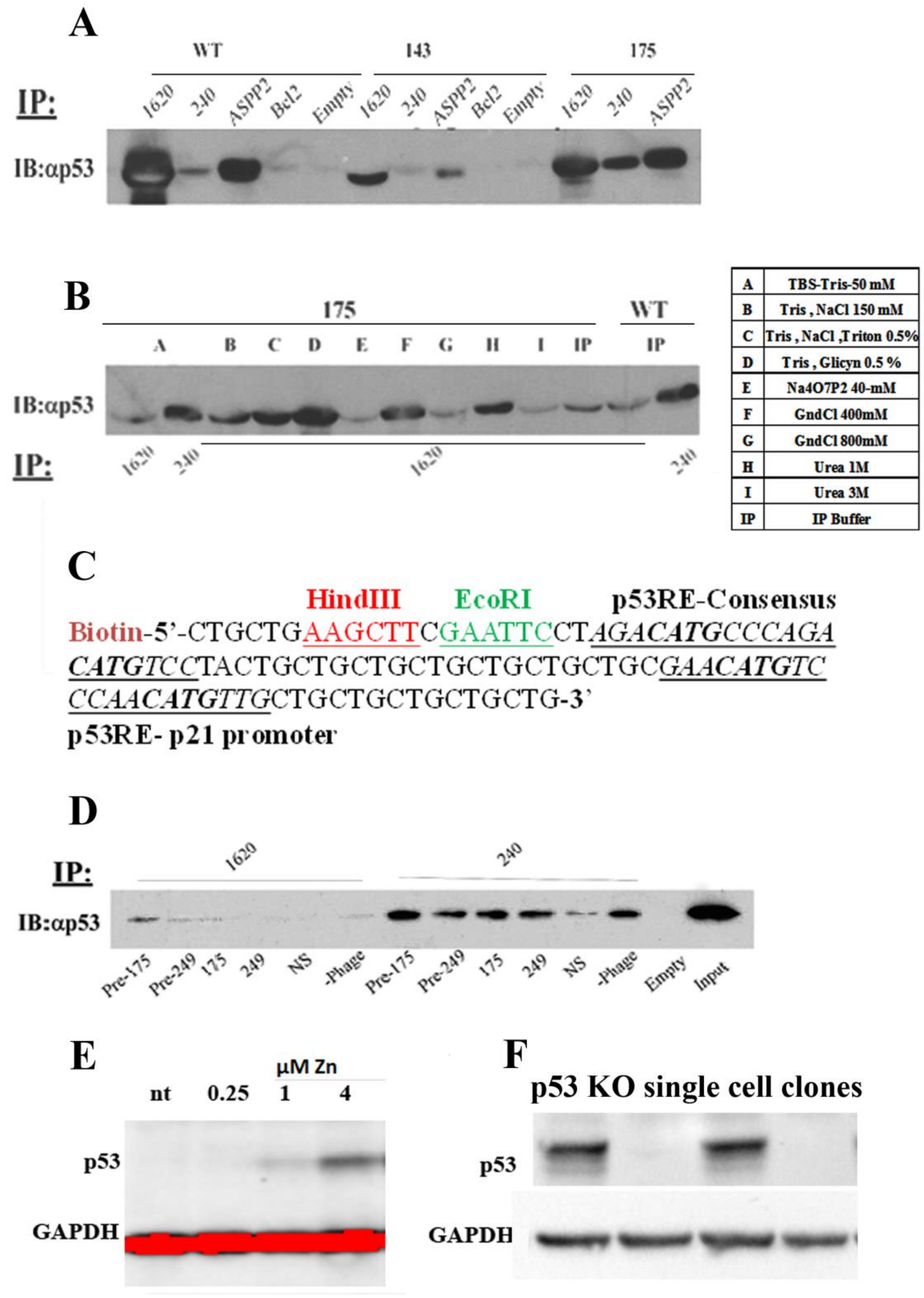
A
WT
143
175
IP:
1620
240
ASPP2
Bcl2
Empty
IB:αp53
B
175
WT
A B C D E F G H I IP IP
IB:αp53
IP:
1620
240
A TBS-Tris-50 mM
B Tris , NaCl 150 mM
C Tris , NaCl ,Triton 0.5%
D Tris , Glicyn 0.5 %
E Na4O7P2 40-mM
F GndCl 400mM
G GndCl 800mM
H Urea 1M
I Urea 3M
IP IP Buffer
C
HindIII
EcoRI
p53RE-Consensus
Biotin-5'-CTGCTGAAGCTTCGAATTCCTAGACATGCCCAGA
CATGTCCTACTGCTGCTGCTGCTGCTGCGAACATGTC
CCAACATGTTGCTGCTGCTGCTGCTG-3'
p53RE- p21 promoter
D
IP:
1620
240
IB:αp53
Pre-175
Pre-249
175
249
NS
-Phage
Empty
Input
E
µM Zn
nt
0.25
1
4
p53
GAPDH
F
p53 KO single cell clones
p53
GAPDH

Figure S1- Calibration of conditions for phage display.

A - Western blot analysis of an immunoprecipitation (IP) experiments, where agarose beads covalently cross-linked to anti-p53 antibodies (PAb1620 or PAb240) or proteins (ASPP2 or Bcl2) were incubated for 3 hours at 4ºC with WT p53, mutp53 R175H or mutp53 V143A proteins (each extracted from Sf9 cells transfected with baculovirus expressing the respective protein). The resultant immunoprecipitates, as well as the supernatant (sup), were subjected to Western blot analysis, using an anti p53 (αp53) antibody conjugated to horseradish peroxidase (HRP).

B - Western blot analysis of an IP experiment, where beads covalently cross-linked to PAb1620 or PAb240 antibodies were incubated with WTp53 or mutp53 R175H for 3 hours at 4°C in various solutions (A-I and IP buffer). The resultant immunoprecipitate, as well as the supernatant (sup), were subjected to Western blot analysis, using an anti p53 (αp53) antibody conjugated to HRP**.** Solution A – 50mM Tris; solution B – Tris, 150 mM NaCl; solution C – Tris, NaCl, 0.5% Triton; solution D – Tris, 0.5% Glycine; solution E – 40mM $Na_4O_7P_2$; solution F – 400mM Guanidine-HCl; solution G – 800mM Guanidine-HCl; solution H – 1M Urea; solution I – 3M Urea; IP – IP Buffer.

C - Sequence of the oligonucleotide used as the binding element for p53**.** The oligonucleotide comprises a 5' biotin label, followed by a HindIII recognition site (underlined), followed by an EcoRI recognition site (underlined), followed by a p53 consensus binding element (underlined, thep53 binding site is composed of two half sites, each of which binds a dimer of p53 and together this forms a complex of DNA and a p53 tetramer), followed by two copies of the p53 response element of the p21 promoter (underlined). For binding experiments, this

oligonucleotide was annealed to a complementary oligonucleotide to form a double stranded (ds) oligonucleotide.

D - Pre-cleared phage induce binding of mutant p53R175H to PAb1620. Beads covalently cross-linked to PAb1620 and PAb240 were incubated with purified mutant p53R175H in the presence of pre-cleared and normal phage from the third selection, non-specific phage (NS), or no phage. Immunoprecipitation was perfomed for 3 hours with beads covalently bound to PAb1620, followed by 3 washing steps. Elution was in sample buffer for 10 minutes at 95°C. The eluted protein was subjected to Western blot analysis using anti p53 (αp53) antibody conjugated to horseradish peroxidase (HRP).

E - Western blot analysis of H1299 cells expressing mutp53 under the control of a Zn^{++} inducible promoter. Cells were treated with different concentrations of Zn^{++} for 24 hours, harvested, lysed and protein levels were determined. 50μg of protein was loaded in each lane.

F - Western blot analysis of single cell clones of ES2 cells stably knocked out for p53 using CRISPR/Cas9. Figure shows 4 representative clones, two clones show a sucssesful knockout (no p53 protein) and two clones in which p53 protein levels were not effected. The later two clones served as controls in viability and p53 target genes expression assays.

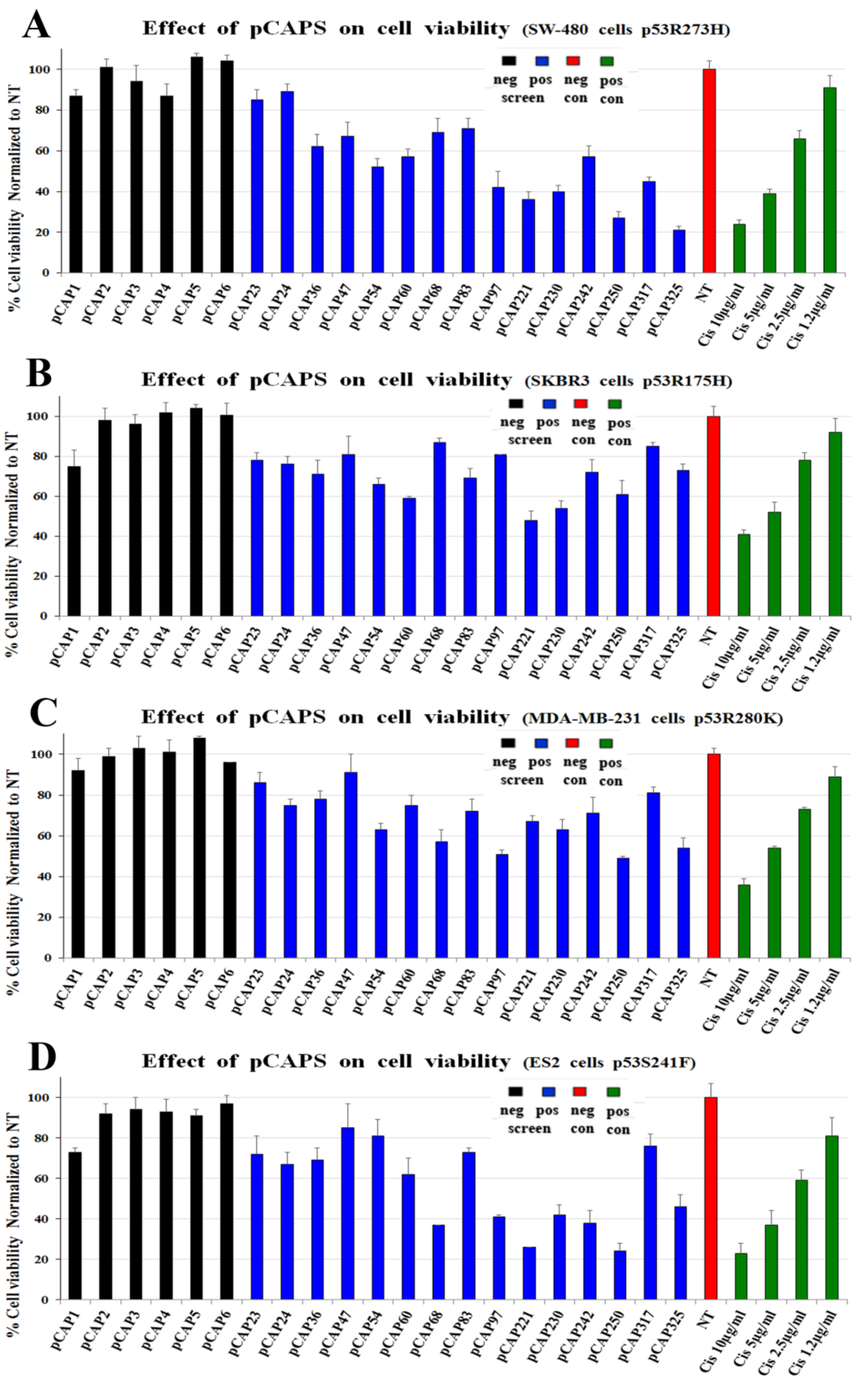
A
Effect of pCAPS on cell viability (SW-480 cells p53R273H)
B
Effect of pCAPS on cell viability (SKBR3 cells p53R175H)
C
Effect of pCAPS on cell viability (MDA-MB-231 cells p53R280K)
D
Effect of pCAPS on cell viability (ES2 cells p53S241F)
neg screen
pos
neg con
pos con
% Cell viability Normalized to NT
0
20
40
60
80
100
pCAP1
pCAP2
pCAP3
pCAP4
pCAP5
pCAP6
pCAP23
pCAP24
pCAP36
pCAP47
pCAP54
pCAP60
pCAP68
pCAP83
pCAP97
pCAP221
pCAP230
pCAP242
pCAP250
pCAP317
pCAP325
NT
Cis 10μg/ml
Cis 5μg/ml
Cis 2.5μg/ml
Cis 1.2μg/ml

Figure S2 – Evaluation of peptides efficacy in different cell lines expressing endogenouse mutp53 forms.

Bar graphs illustrating the effect of various selected peptides on cell viability for the indicated cell lines expressing endogenouse mutp53. Black bars represent randomly screened peptides, blue bars represent peptides with a significant effect on cell viability, red bars are negative controls (non-treated) and green bars are positive controls (increasing concentrations of cytotoxic drug). Cell lines were treated with selected peptides, Cis-platinum was used as positive control for cell death. 48 hours after treatment, cells were washed with PBS, and the remaining attached cells were stained with Crystal violet and washed 4 times with PBS. Stained cells were dissolved in 10% acetic acid and plates were taken for optical density measurement at 595nM.

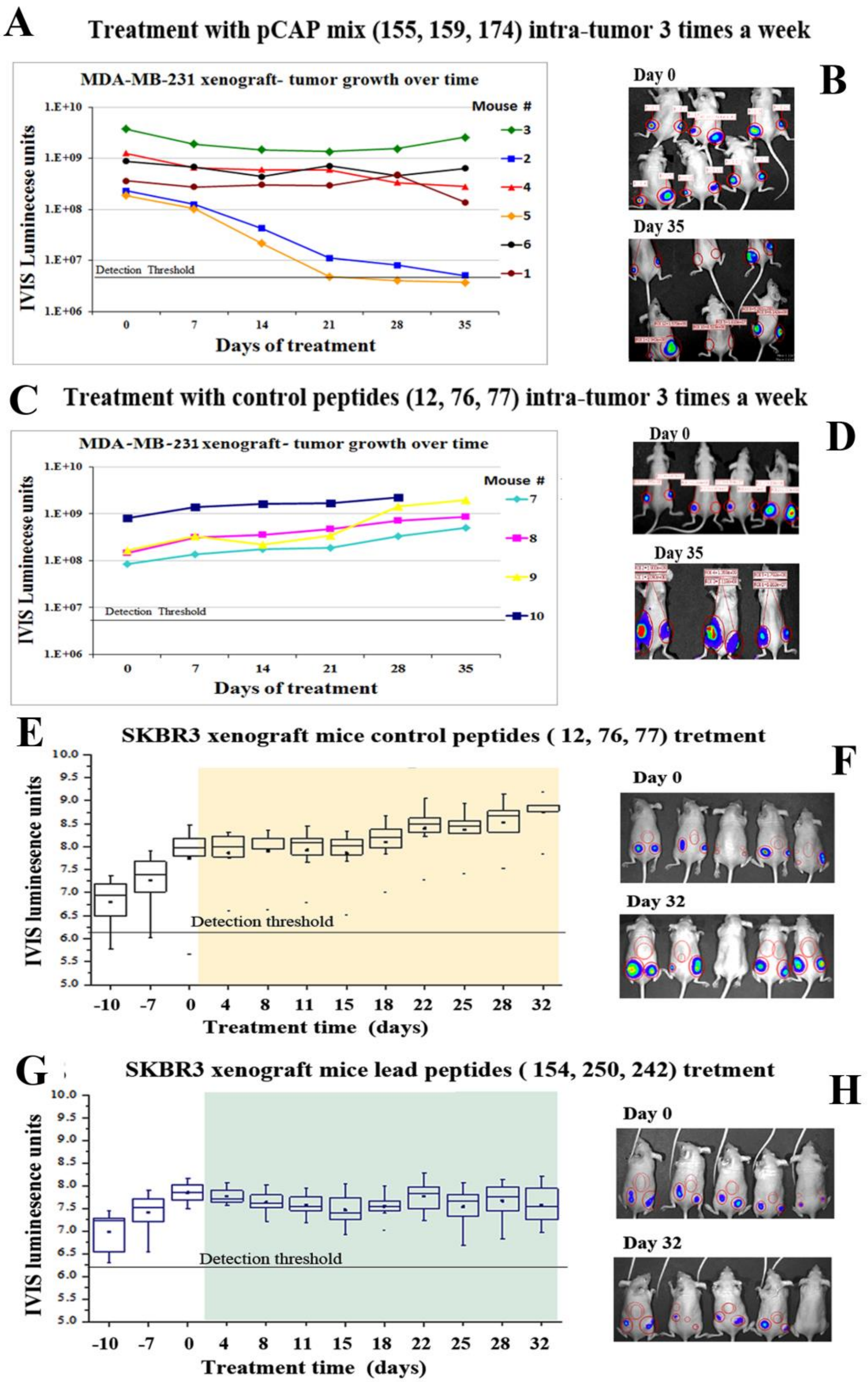
A
Treatment with pCAP mix (155, 159, 174) intra-tumor 3 times a week
MDA-MB-231 xenograft- tumor growth over time
IVIS Luminecese units
1.E+10
1.E+09
1.E+08
1.E+07
1.E+06
Mouse #
3
2
4
5
6
1
Detection Threshold
0
7
14
21
28
35
Days of treatment
Day 0
Day 35
B
C
Treatment with control peptides (12, 76, 77) intra-tumor 3 times a week
MDA-MB-231 xenograft- tumor growth over time
IVIS Luminecese units
Mouse #
7
8
9
10
Detection Threshold
Days of treatment
Day 0
Day 35
D
E
SKBR3 xenograft mice control peptides (12, 76, 77) tretment
IVIS luminesence units
Detection threshold
-10
-7
0
4
8
11
15
18
22
25
28
32
Treatment time (days)
F
Day 0
Day 32
G
SKBR3 xenograft mice lead peptides (154, 250, 242) tretment
IVIS luminesence units
Detection threshold
Treatment time (days)
H
Day 0
Day 32

Figures S3 - The in vivo effect of peptides in a mouse triple negative breast cancer xenograft model (MDA-MB-231 cells)

A-D 10^6 MDA-MB-231 cells expressing endogenous mutp53 and stably expressing luciferase were injected into the hips of nude mice. When tumors reached visible size, mice were treated by intra-tumoral injection, three times a week, with either a mixture of 3 control peptides that showed no phenotype in vitro (pCAPs 76, 77 and 12; 2µg of each peptide) or a mixture of 3 test peptides that exhibited mutp53-reactivating ability (pCAPs D60R, 24R and 174; 2µg of each peptide).

Luciferase readings of MDA-MB-231 tumors in control peptide-injected mice (**A**) and in the effective pCAP mix group (**B**) (logarithmic scale) as a function of time after initiation of treatment (peptide injection). Mice 2 and 5 showed a complete response, reaching bioluminescence readings that were as low as or close to the background threshold detection levels of the IVIS system ($5x10^6$ photons) even after 21 days of treatment. Following cessation of the treatment after 35 days, mice numbers 2 and 5 were kept alive and monitored for an additional 21 days; no reappearance of tumors was detected either visually or by live imaging.

C - Live imaging of the control group of MDA-MB-231-injected mice, performed at termination of the experiment (day 35)

D - Live imaging of MDA-MB-231-injected mice treated with the effective peptides mix, performed at termination of the experiment (day 35)

E,F - 10^6 SKBR3 breast cancer cells, expressing endogenous mutp53 and stably expressing luciferase, were injected into the hips of nude mice. When tumors reached visible size, mice were treated by intra-tumoral injection, three times a week, with either a mixture of 3 control

peptides that showed no phenotype in vitro (pCAPs 76, 77 and 12; 2μg of each peptide) or a mixture of 3 test peptides that exhibited mutp53-reactivating ability in culture (pCAPs 154, 250 and 242; 2μg of each peptide).

E (control group mice) and **F** (effective pCAP mix group) are depicted on a logarithmic scale box-plot displaying the luciferase readings in tumors as a function of time. Average (horizontal line), standard deviation (box), highest and lowest reads (eror bars) are shown, before (until day 0; white bacground) and after initiation of treatment (peptide injection; colored backgrond).

G - Live imaging of control group mice at the beginning of treatment (day 0) and termination of the experiment (day 32)

H - Live imaging of mice treated with an effective peptides mix at the beginning of treatment (day 0) and at the termination of experiment (day 32)

Table S1 - Calibration of selection strategies and titration.

- Panning of NEB phd-12 phage display library against $p53^{R249S}$ (Table S1A) and against $p53^{R175H}$ (Table S1B), using the PAb1620+mutp53 strategy alone compared to dual strategy panning in alternating rounds of selection (Table S1C), where panning in the first round was performed with PAb1620Ab+mutp53. The second round of panning was for phage binding imobilized p53RE DNA-WTwtp53 complex, and the third round was employed PAb1620Ab+mutp53 selection again. The phage titer shows an increase in eluted phage between selection rounds in all performed experiments. However, the control panning experiment, performed by panning only against PAb1620 (without p53) in the third rounds, indicates that the use of a single selection strategy results in a high degree of background (phage binding directly to PAb1620) regardless of p53. The dual selection strategy (Table S1C), on the other hand, yeilded a significantly higher number of phage binding to the p53+PAb1620 complex compared to PAb1620 alone.

Table S1A- selection with R249S:

**Selection round**	**The selection marker**	**Number of eluted phage** (Phage/µl)
**1**	1620Ab + FL-p53R249S	10^3
**2**	1620Ab+ FL-p53R249S	10^5
**2**	1620Ab	10^5
**3**	1620Ab+ FL-p53R249S	10^6
**3**	1620Ab	$3*10^6$

Table S1B- selection with R175H:

**Selection round**	**The selection marker**	**Number of eluted phage** (Phage/µl)
**1**	1620Ab + FL-p53 H175R	10^3

**2**	1620Ab+ FL-p53 H175R	10^5
**2**	1620Ab	10^5
**3**	1620Ab+ FL-p53 H175R	10^6
**3**	1620Ab	$2*10^6$

Table S1C- Alternating selection with mutp53 and wtp53

**Selection round (Phd-12)**	**The selection marker and the p53 form**	**Number of eluted phage (Phage/µl)**
**1**	1620Ab + p53R249S	$3.5*10^2$
**1**	pAb1620 + p53R175H	$2*10^2$
**2**	p53RE + wtp53	$2*10^3$
**3**	pAb1620 + p53R175H	10^5
**3**	pAb1620 + p53 R249S	10^5
**3**	pAb1620	$4*10^3$

Table S2 – Performed phage display selections: strategies and titration of selected eluetes.

#	Library	round	Selection type	Tither
1	**phd-7**	**1°**	1620 + 175	**$2*10^2$**
31	**phd-7**	**1°**	1620--wt	**$2*10^3$**
32	**phd-7**	**1°**	Tag--wt	**$1*10^3$**
81	**phd-12**	**1°**	Tag--wt	**$5*10^3$**
4	**phd-7**	**1°**	RE--wt	**$1*10^3$**
33	**phd-7**	**2°**	1620+175, 1620-wt	**$2*10^5$**
39	**phd-7**	**2°**	1620+175, 1620+175	**$1.5*10^4$**
47	**phd-7**	**2°**	1620+175, 1620+175	**$2*10^4$**
45	**phd-7**	**2°**	1620+175, 1620-175	**$2*10^4$**
52	**phd-7**	**2°**	Tag-wt, 1620+175	**$1.5*10^6$**
41	**phd-7**	**2°**	1620+175, 1620	**$8*10^3$**
90	**phd-12**	**2°**	Tag-wt, 1620+175	**$1.5*10^5$**
34	**phd-7**	**2°**	1620+175, Tag-wt	**$5*10^4$**
40	**phd-7**	**2°**	1620+175, Tag+175	**$3*10^4$**
48	**phd-7**	**2°**	1620+175, Tag+175	**$4*10^4$**
40	**phd-7**	**2°**	1620-wt, Tag+175	**$4*10^4$**
44	**phd-7**	**2°**	1620+175, Tag	**$1*10^3$**
51	**phd-7**	**2°**	1620-wt, Tag+175	**$2*10^6$**
83	**phd-7**	**2°**	Tag-wt, Tag+175	**$2*10^6$**
55	**phd-12**	**2°**	1620+175, Tag-wt	**$2*10^4$**
5	**phd-7**	**2°**	1620+175, Ni-wt	**$3*10^4$**
82	**phd-7**	**2°**	Tag-wt, Ni-wt	**$3*10^3$**
10	**phd-12**	**2°**	1620+175, Ni-wt	**$1*10^5$**
38	**phd-7**	**2°**	1620+175, RE-wt	**$5*10^5$**
53	**phd-12**	**2°**	1620+175, RE-wt	**$5*10^4$**
86	**phd-7**	**2°**	Tag-wt, RE-wt	**$2*10^5$**
91	**phd-12**	**2°**	Tag-wt, RE-wtDBD	**$1*10^5$**
35	**phd-7**	**3°**	1620+175, Ni-wt, 1620-wt	**$3*10^5$**
42	**phd-7**	**3°**	1620+175, Ni-wt, 1620+175	**$5*10^4$**
64	**phd-7**	**3°**	1620+175, RE-wt, 1620+175	**$1*10^6$**

#	Library	round	Selection type	Tither
36	**phd-7**	**3°**	1620+175, Ni-wt, Tag-wt	**$1*10^6$**
43	**phd-7**	**3°**	1620+175, Ni-wt, Tag+175	**$2*10^6$**
65	**phd-7**	**3°**	1620+175, RE-wt , Tag+175	**$2*10^6$**
69	**phd-7**	**3°**	Tag-wt, 1620+175, Tag+175	**$5*10^6$**
85	**phd-12**	**3°**	1620+175, RE-wt, Tag+175	**$2*10^5$**
92	**phd-7**	**3°**	1620+175, Ni-wt, Tag-wtDBD	**$3*10^5$**
93	**phd-7**	**3°**	1620+175, Ni-wt, Tag+175	**$4*10^5$**
94	**phd-7**	**3°**	1620+175, Ni-wt, Tag+249DBD	**$4*10^6$**
95	**phd-7**	**3°**	Tag-wt, 1620+175, Tag+175	**$5*10^6$**
98	**phd-7**	**3°**	1620-wt,Tag+175, Tag+249DBD	**$5*10^6$**
37	**phd-7**	**3°**	1620+175, Ni-wt, RE-wt	**$5*10^5$**
24	**phd-7**	**3°**	1620+175, Ni-wt, RE	**$5*10^2$**
57	**phd-12**	**3°**	1620+175, Ni-wt, RE-wt	**$8*10^4$**
75	**phd-7**	**3°**	Tag-wt, 1620+175, RE-wt	**$5*10^4$**
96	**phd-7**	**3°**	Tag-wt, 1620+175, RE+wtDBD	**$1.5*10^6$**
97	**phd-12**	**3°**	1620+175, Tag-wt, RE+wtDBD	**$5*10^4$**
118	**phd-12**	**3°**	RE+249DBD	**$5*10^4$**
63	**phd-7**	**4°**	1620+175, Ni-wt, RE-wt, 1620+175	**$1*10^6$**
71	**phd-12**	**4°**	1620+175, Ni-wt, RE-wt, 1620+175	**$1*10^6$**
66	**phd-7**	**4°**	1620+175, Ni-wt, Tag+175, 1620+175	**$3*10^6$**
62	**phd-7**	**4°**	1620+175, Ni-wt, Tag-wt, 1620+175	**$3*10^6$**
84	**phd-12**	**4°**	1620+175, Ni-wt, RE-wt, Tag+175	**$5*10^5$**
87	**phd-7**	**4°**	Tag-wt, 1620+175, RE-wt, Tag+175	**$9*10^6$**
102	**phd-7**	**4°**	Tag-wt, 1620+175, RE-wt, Tag	**$4*10^3$**
121	**phd-7**	**4°**	RE	**$1*10^4$**
112	**phd-7**	**4°**	RE-wtDBD	**$5*10^4$**
113	**phd-7**	**4°**	RE-249DBD	**$6*10^6$**
96	**phd-7**	**4°**	RE+wtDBD	**$1.5*10^6$**
101	**phd-7**	**4°**	RE+wtDBD	**$1.5*10^6$**
97	**phd-12**	**4°**	RE+wtDBD	**$5*10^4$**
117	**phd-12**	**4°**	RE+249DBD	**$1.5*10^6$**

Table S3 - Summary of peptide functional screens and sequence similarities

The results of a variety of functional assays performed on representative synthetic peptides, presented as a heat map. Peptides were evaluated for their effect in each assay and assigned a score and corresponding color according to performance relative to a positive control (score 0- no effect-white – score 6- at least as effective as the positive control- black). Assays include: mutp53 conformation (orange), mutp53 DNA binding (red), viability of mutp53-expressing cells and induction of p53 target genes as assayed by by qRT-PCR (blue). Peptide sequences are aligned and have a background color according to similarity to known p53 interacting proteins and to each other. Since peptide sequences have been changed by us along time in an attempt to yield more effective sequences according to performance in the different assays, table 2 also reflects the "evolutionary" process of peptide analysis. First set of peptides tested (pCAPs 1-160) comprised sequences identified in the phage display selection. Later on, lead sequences were modified to resemble p53 interacting proteins. Later, arginine residues and N-terminal myristoil were added to enhance cell permeability (pCAPs 200-326). Highlighted amino acids are similar to motifs found in p53-interacting proteins.

			Conform			DNA		Viabiliy								PCR		
Name	***Peptide Sequence***	***Similarity***	H1299	SK3	BT549	H1299	SK3	H1299	HOP92	ES2	SK3	MDA	wi48	EP156	H23	MDA231	ES2	SCORE
pCAP-1	**EVTFRHS VV**	**p53**			1	1		1	3	4	3				1	3	2	19
pCAP-2	VWVHDSCHANLQNYRNYLLP									2								2
pCAP-3	FWTQSIKERKM LNEHDFEVR							1	1	2								4
pCAP-4	EHDFEVRGDVVNGRNHQGPK									2								2
pCAP-5	LEVIYM I									2								2
pCAP-6	LGIDEDEETETAPE									2		2						4
pCAP-7	SPLQTPAAPGAAAGPALSPV					1							2					3
pCAP-8	LTFEHYWAQLTS																	0
pCAP-9	**TIIHREDEDEIEW**		2			3			3	2	2			3				15
pCAP-10	VAEFAQSIQSRIVEWKERLD						3											3
pCAP-11	**EWKERLDKEFS LS VYQ KMKF**						3	3	3	5	2							16
pCAP-12	GGGGGGGGGGGG																	0
pCAP-13	**HFS HHLK**																	0
pCAP-14	**HHFS HHWKT**		2					2	2		3							9
pCAP-15	THFSHHLKH		2					2	2		3			3		3		15
pCAP-16	L**PNPPER**HH		2			3	3	3	3	4				4				22
pCAP-17	LHSKTLVL		2		2	3	3			3	3			4				20
pCAP-18	**S HQ VHTHHNN**			3									3					6
pCAP-19	**NPNTYVPHWMRQ**																	0
pCAP-20	**HHPWTHHQ RWS**	**CHD1**	2	4		2					3				4		4	19
pCAP-21	NHPWQFPNRWTV		4	2	2	2			2		3	3		4			3	25
pCAP-22	**S LLIGFGIIRS R**																	0
pCAP-23	**IRILMFLIGCGR**		2		2	1		3	3	3	3			4	4			25
pCAP-24	**LRCLLLLIGRVG**		3	2	2	3	3			3	3			4				23
pCAP-25	YRRLLIGM M W																	0
pCAP-26	DEFHSFYTARQTG																	0
pCAP-27	**VHEVTHHWL**			2								3						5
pCAP-28	HTDSHPHHHHPH		2			2				3	3	3		4		2	2	21
pCAP-29	**KPDS PRV**																	0
pCAP-30	TIHPSIS		3		2	3	2	2	2	2							2	18
pCAP-31	**PPYS Q FLQ WYLS**																	0
pCAP-32	**TS PLQS LK**																	0
pCAP-33	SILTLRLRRLRR		2	2						4		4		4				16
pCAP-34	**S ILTLS RIVLGWW**									2	4	4		4				14
pCAP-35	GAM HLPWHM GTL		2	2	2		4	3	2	3	3		3	4	2		3	33
pCAP-36	YPTQGHLR		5	3				2	2	3	3		3	5	3		3	32
pCAP-37	**KLQ VPIK**											2						2
pCAP-38	WTLSNYL											2						2
pCAP-39	**DS LHS TY**											2						2
pCAP-40	**S EFPRS WDMETN**																	0
pCAP-41	**WHHRQ QIPRPLE**											2						2
pCAP-42	**MHPPDWYHHTPKH**		3	2		4		2	2	3	3	3		3			3	28
pCAP-43	**TDS HS HH**									2	3	3	3					11
pCAP-44	**VPHIHEFT**		2	3			3			3	2							13
pCAP-45	**HDTHNAHVG**																	0
pCAP-46	**AS WQ ALALYAAGW**		2		2		2			2	3		3	4	6	3	2	29
pCAP-47	**TLYLPHWHRH**		2				3				3		3					11
pCAP-48	IPM NFTSHSLRQ		2		2			3	3	3								13
pCAP-49	**TRILCIVMM**									5			3					8
pCAP-50	**WS EYDIPTPQ IPP**																	0
pCAP-51	**AILTLILRRVIWP**							2	2									4
pCAP-52	**S PYPIRT**													3				3
pCAP-53	**ILVIIQ RIM**											3						3
pCAP-54	**IRGRIIR**		3		2	2					4	4	4	4				23
pCAP-55	**FLLPEPDENTRW**													4				4
pCAP-56	**ATPFHQT**																	0
pCAP-57	**LMS NAQY**					2	2							4				8
pCAP-58	**S ILPLFLIRRS G**																	0
pCAP-59	FLIRRSG																	0
pCAP-60	**SFILFIRRGR**LG			2	1	3		4		3	5		2		4		2	26
pCAP-61	**KDLPFYS HLS RQ**		2	3				2	2	5			3					17
pCAP-62	YELPHHAYPA									5		2	3					10
pCAP-63	**HNHH**HSQ**HTP**QH		2		3						2	2						9
pCAP-64	APSIFTPHAWRQ											2						2

			Conform			DNA		Viabiliy								PCR		
Name	***Peptide Sequence***	***Similarity***	H1299	SK3	BT549	H1299	SK3	H1299	HOP92	ES2	SK3	MDA	wi48	EP156	H23	MDA231	ES2	SCORE
pCAP-65	**THFS HHLK**HRRRRRRRRRRRR			2		2		2			2	4	3					15
pCAP-66	**THFS HHLKG**GGRKKRRQRRRP																	0
pCAP-67	**LHS KTLVL**GGGRRRRRRRGDR																	0
pCAP-68	YRRLLIGMM WRRRRRRRRRRRR		4	5		4		3	5	5		4	2			2	3	37
pCAP-69	**SILTLS RR**RRRRRRRRR																	0
pCAP-70	SILTLSRGRKKRRQRRRR		3	3		2		2	3		3	3	3		2	2	4	30
pCAP-71	WTLSNYLGGRKKRRQRRRR		2															2
pCAP-72	**SCRCRLR**RRRRRRRRR								2			3						5
pCAP-73	**SCRCRLR**GDRGDR																	0
pCAP-74	IRGRIIRRKKRRQRRRRGDR								3	3								6
pCAP-75	**TRILCIV**RKKRRQRRRRGDR		3	3		2		2	5	5	3	4	2			3	3	35
pCAP-76	GGGGGGGGGRRRRRRRRR																	0
pCAP-77	SEYLCSSLDAAG																	0
pCAP-78	GESFVQHVFRQN																	0
pCAP-79	SVHHHHRMHLVA																	0
pCAP-80	**HLHKHH**YKDSRM		3			3						3						9
pCAP-81	VRCIFRGIWVRL								2									2
pCAP-82	QIPHRSSTALQL							3	3									6
pCAP-83	HSS **HHH**PV**HS WN**		4	2		5	2	4			4		3	3		3		30
pCAP-84	GRRRFCM																	0
pCAP-85	KLTIHHH																	0
pCAP-86	FGSHHEL																	0
pCAP-87	HANLHHT		3	2		2		3	3	4		4	3			4	4	32
pCAP-88	SYQTMQP			2								4						6
pCAP-89	SILTLSCRCRLRLWR			2						4		2						8
pCAP-90	SCRCRLR					2		4	4	2								12
pCAP-91	STTHIHA					4					3							7
pCAP-92	**FPHLVS SLTT**								4		3							7
pCAP-93	**ATQHHYIK**		4	4				2			3							13
pCAP-94	LRFIDYP		4															4
pCAP-95	**HQIHRNHTY**							4	4									8
pCAP-96	**GTVDHHA**																	0
pCAP-97	**WNHHHS TPHPAH**		1	3		4	2	3	4	5		3	3	3		3	5	39
pCAP-98	**HS SGHNFVLVRQ**											2						2
pCAP-99	**GLHLFTTDRQGW**							2	3			2						7
pCAP-100	**FPGHTIH**			5				3		3	2	3				3		19
pCAP-101	**IRFILIR**									3								3
pCAP-102	SSVHHRG									3								3
pCAP-103	LRRQLQL									3								3
pCAP-104	**LLRLGLI**		3		3	6				3	3	3					3	24
pCAP-105	**S RIVLGW**		3	2														5
pCAP-106	LIRRCSLQR		3	5														8
pCAP-107	DRLSVFLFIM																	0
pCAP-108	**IIRGNFLIGGRL**		3			3			2	3	3	3				3	3	23
pCAP-109	GPIKHHLQHH								2			2						4
pCAP-110	LFILVFR											2						2
pCAP-111	**S NIHHQV**									3		2						5
pCAP-112	TTSHHPK											2				3		5
pCAP-113	**HTTAHTH**												3					3
pCAP-114	AISHHTR																	0
pCAP-115	**HPHNHTVHNVVY**								3									3
pCAP-116	**KHHPFDHRLGNQ**																	0
pCAP-117	**DHS KFVPLFVRQ**		3															3
pCAP-118	SNHHHRH**HTNTH**			2		2					3	3	3			3		16
pCAP-119	**HS AHHTM**																	0
pCAP-120	**S IRTLGRFLIIRV**									3								3
pCAP-121	LTLMRLRIIG								2	3								5
pCAP-122	HSYSPYYTFRQH		2							3								5
pCAP-123	GLCRIIL									3								3
pCAP-124	VM VLFRILRGSM							2										2
pCAP-125	ELGLHRH																	0
pCAP-126	RRLRICV																	0
pCAP-127	SPPIRHH		3															3
pCAP-128	CILRLWW		3	2		3			3	3		3	2			3		22
pCAP-129	FRSFAIPLVVPFRRRRRRR						4	3	2	2								11

			Conform			DNA		Viabiliy								PCR		
Name	***Peptide Sequence***	***Similarity***	H1299	SK3	BT549	H1299	SK3	H1299	HOP92	ES2	SK3	MDA	wi48	EP156	H23	MDA231	ES2	SCORE
pCAP-130 (1R)	**EVTFRHS VVR**RRRRRRRRRR	p53					6	3	4									13
pCAP-131 (16R)	**LPNPPER**HHRRRRRRRRRRR		3		3	3	3	2	3	1								18
pCAP-132 (21R)	NHPWQFPNRWTRRRRRR						3	4										7
pCAP-133 (22R)	SLLIGFGIIRSRRRRRRRR							2	2	6								10
pCAP-134 (23R)	**IRILMFLIGCG**RRRRRRRR				4		3	2	6	6	3	3		3	3	3		36
pCAP-135 (28R)	HTDSHPHHHHPHRRRRR						3	3	2	2								10
pCAP-136 (33R)	SILTLRLRRLRRRRRRRR						3	3	5	3								14
pCAP-137 (35R)	GA**MHLPWHMG**TRRRRRR							3	3	2								8
pCAP-138 (36R)	YPTQGHLRRRRRRRRRRR						3	4	2	2								11
pCAP-139 (42R)	MHPPDWYHHTPKRRRRRR						2	3	2	2		3						12
pCAP-140 (43R)	TDSHSHHRRRRRRRRRRR							3	3									6
pCAP-141 (46R)	SWQALALYAAGWRRRRRR						6	3		5	3	3	3	3	3			29
pCAP-142 (47R)	TLY**LPHWHRHR**RRRRRRRRR						6	2		3				3				14
pCAP-143 (48R)	IPMNFTSHSLRQRRRRRRRRR						2			4								6
pCAP-144 (60R)	S**FILFIRRG**RLGRRRRRRRRR		2	2		2	2	2	3	3	3	2	6	6		4	4	41
pCAP-145 (83R)	HS**S HH**HPVH**S WN**RRRRRRR						3	2		2								7
pCAP-146 (87R)	**HANLHHT**RRRRRRRRRRR						3	2	3	3								11
pCAP-147 (93R)	ATQHHYIKRRRRRRRRRRR						4	3		3								10
pCAP-148 (97R)	WNHH**HS TPHPD**RRRRRRRRR		4	4			4	5	3	4	2		3	3	3	4	4	43
pCAP-149 (100R)	F**PGHTIHRR**RRRRRRRRR						3	5	3	2								13
pCAP-150 (108R)	IIRG**NFLIGGRL**RRRRRRRRR		4	4			4	3		2	5							22
pCAP-151 (128R)	CILRLWWRRRRRRRRRRR						5	4	5	4	3	3		3	3	3		33
pCAP-152 (25R)	**YRRLLIGM**RRRRRRRRRRR						5	3	3									11
pCAP-153	YWSAPQPATRRRRRRRRRRR						3		3									6
pCAP-154 (24R)	LRCLL**LLIGRVGRK**KRRQRR		6	4	5		4	5	5	5	3	5	3	4	5	3		57
pCAP-155 (54R)	**IRGRIIR**RRRRRRRRR			2	2	5	5	5		4	4	4	4		3			38
pCAP-156 (44R)	VPHIHEFTRRRRRRRR																	0
pCAP-157 (60TAT	**SFILFIRRGR**LGRKKRRQRRRP		1	1	1	2	3	3	3	3	5	2	2	5	2	4		37
pCAP-158 (60RGD	**SFILFIRRGRL**GRGDR		1	1			4		4		4		1	1				16
pCAP-159	**Fitc-SFILFIRRGRL**GRRRRRRR		1		1			4	4		4			4	3	3		24
pCAP-160 (D60R)	rrrrrrrglrg**rriflifs**		1	1	1		3	5	4	4		5	4	5	4	5	4	46
pCAP-161	**HNAH**		3	2		2	2	3	5	3	5		4		4			29
pCAP-162	**SILT**			2														2
pCAP-163	**LTLS**				4													4
pCAP-164	**PLTLI**																	0
pCAP-165	**SLLIG**																	0
pCAP-166	**KPPER**																	0
pCAP-167	**FILIR**									5								5
pCAP-168	**CRIIR**																	0
pCAP-169	**SFILI**																	0
pCAP-170	**IRILM**			5	5	5	3	3		3	3							27
pCAP-171	**PHHHS**																	0
pCAP-172	**EFHS**																	0
pCAP-173	**RLRRL**																	0
pCAP-174	**HSTPHPD**		4	4	5	4	5	4	5	4	4	5	1	4	4			53
pCAP-175	**DSPR**																	0
pCAP-176	**HPWTH**																	0
pCAP-177	**HFSHH**																	0
pCAP-178	**RRVI**																	0
pCAP-179	**ILVI**																	0
pCAP-180																		0

			Conform			DNA		Viabiliy								PCR		
Name	***Peptide Sequence***	***Similarity***	H1299	SK3	BT549	H1299	SK3	H1299	HOP92	ES2	SK3	MDA	wi48	EP156	H23	MDA231	ES2	SCORE
pCAP-201	**HPTHPIR**LRDNLTR	14-3-3		3														3
pCAP-202	DEDAK**FRIRILMR**R	APAF1		2	3			3	2		3							13
pCAP-203	RRRHDSCHNQLQ**NYDHSTE**	ASPP1	3		3		3	4	4	6	4		6	5	4	4		43
pCAP-204	MSTESN**MPRLIQN**DDRRR	ASPP2					3	4		4			5			5		21
pCAP-205	R**CRNRKKEKT**ECLQKESEK	ATF3		2	3													5
pCAP-206	RR**RSHSQENVD**QDTDE	BAK			2		3	3	3	2		1	5			5		24
pCAP-207	RR**SRSNE**DVEDKTEDE	BAK																0
pCAP-208	RRIRSGGKDHA**WTPLHENH**	BARD1																0
pCAP-209	**HTPHPP**VARTS**PLQT**PRR	BCL2																0
pCAP-210	DEFE**RYRRFSTS**RRR	BCL-X	1		1	2				2			5	3	3	6		23
pCAP-211	PDS**EPPRMELR**RR	BCR																0
pCAP-212	myr-REEE**TILIIRR**R	BRG1			2													2
pCAP-213	RRIK**MIRTSESFIQH**IVS	BTF			1		2											3
pCAP-214	RRRESEQ**RSISLHHHS**T	C-ABL						2	2									4
pCAP-215	RRDTF**DIRILMAF**	CARM1																0
pCAP-216	myr-**HFNHYTF**ESTCRRRRC	CAS		2														2
pCAP-217	**HSTPHPPQPPER**RR	CCDC8						2	2									4
pCAP-218	RREVTEL**HHTHEDR**R	CEP72																0
pCAP-219	SY**RHYSDH**WEDRRR	CEID2									1							1
pCAP-220	**STTHPHP**GTSA**PEP**ATRRR	CHD6	2					2			2							6
pCAP-221	myr-RRK**HNKHRPEPD**SDER	CTF2		3	3	3	3	2	5	6	5	3	6	5	6	4	4	58
pCAP-222	RYEENNG**VNPPVQ**VFESRTR	CUL7			4													4
pCAP-223	**SPWTHER**RCRQR	CYP27B1																0
pCAP-224	RRKSE**PHSLSG**GYQTGAD	DIABLO						2	2									4
pCAP-225	**HTIHSISDFPEP**P**DR**RRR	DMP1									3							3
pCAP-226	DDSDN**RIIRYRR**	G3BP2						3	3		3							9
pCAP-227	myr-RR**KILFIRLMHNKH**	GAS2	3	1	1	5	1	2	6	5	6	3	6	6	3	5		53
pCAP-228	DEDAA**HSTGHPHN**SQHRRRR	HIPK1				3												0
pCAP-229	myr-PRV**LPSPHTIHPSQY**P	HIPK2		2		4	3	4	4		3		3	3	3	3	3	35
pCAP-230	R**KRGK**SYAFF**VPPSESKE**RW	HMGB	2	1	2	4	3	5	5	4	4	1	6	4	6	6		53
pCAP-231	HRTQ**STLILFIRR**GRET	HTRA2	3		6													3
pCAP-232	RS**RSSHLRDHE**RTHT	HZF																0
pCAP-233	SHY**HTPQNPPS**TRRR	IFI16	3				3	3	3		3							15
pCAP-234	HRTGHYTRC**RQRCRSRSHNR**[illegible]	KLF4									2							2
pCAP-235	RS**YSKLLCLLER**LRISP	MIF		2	5			2	2		2							13
pCAP-236	RRR**STNT**FLGEDFDQ	MORT																0
pCAP-237	**HTIHVH**YPGNRQ**PNPPL**ILQR	MULE		3	2	5					2							12
pCAP-238	**TSPHP**SLPRHIYPRR	NFAT	2			4												6
pCAP-239	REGFYG**PWHEQR**RR	OGA	2								2							4
pCAP-240	TE**QHHYIPH**RRR	OSGIN2									3							3
pCAP-241	**LIGLSTSP**RPRIIR	PAR3																0
pCAP-242	myr-RRLIV**RILKLPNPPER**	PARC	2		4		4	5	5	5	5	3	4	6	6	5	5	55
pCAP-243	RRCR**SILPLLLLSR**	PERP					2											2
pCAP-244	RRVSELQ**RNKHGR**KHEL	PIAS1			3			2	2									7
pCAP-245	N**HITN**GGEEDSDCS**SRRRR**L	PIN1			3		3	2	2									10
pCAP-246	RRRLDDEDV**QTPTPS**EYQN	PIRH2			3			2										3
pCAP-247	RRITE**IRGRTGK**TTLTYIED	RAD51			3		3											6
pCAP-248	EIYGES**GKTDEH**ALDTEYRR	RAD51																0
pCAP-249	myr-DERTGKT**RRYIDTRDIR**R	RAD51					3				3							6
pCAP-250	myr-R**RHSTPHPD**	RAD9	1	3	2	3	5	6	6	5	6	3	6	5	5	4	4	64
pCAP-251	R**LRRVILRS**YHE	RAD9						3	3									6
pCAP-252	**RRVILR**SYDGG**HSTPHPD**	RAD9																0
pCAP-253	TGKTFV**KRHLTE**FEKKYR	RAN																0
pCAP-254	NHFDYDTIELDTAGEYSRRR	RAS																0
pCAP-255	D**PEPPRY**LP**PPPER**R	RASSF5																0
pCAP-256	RTLHG**RRVIL**HEGG**HSISD**LK	RPA70									2							2
pCAP-257	myr-H**SSHHHPT**VQHRR	SIN3A						4			4							8
pCAP-258	**FLIGPDRLIR**SR	SIVA				5					4		5	2	2	4		22
pCAP-259	myr-R**TLIGIIR**SHHL**TLIRR**	SMG1			4	5	4						5	3	2	2		21
pCAP-260	RR**TFIRHRI**DSTEVIYQDED	STK11																0
pCAP-261	RRRQ**PLP**SA**PEN**EE	STK15						2		3								0
pCAP-262	ESKTGHKSEEQRLRRYR	TBP																0
pCAP-263	YDDE**HNHHPHHST**HRRR	TSC22																0
pCAP-264	RRRREV**HTIHQHGIV**HSD	TTK																0

Name	Peptide Sequence	Similarity	Conform H1299	Conform SK3	Conform BT549	DNA H1299	DNA SK3	Viabiliy H1299	HOP92	ES2	SK3	MDA	wi48	EP156	H23	PCR MDA231	ES2	SCORE
pCAP-265	EEPDRQPSGKRGGRKRRSR	TWIST						3										0
pCAP-266	HHRLSYFIVRRHSTHASR	TWIST						2										0
pCAP-267	KRGGRKRRGGG**HRLSYFIR**R	TWIST	2		4		6			2	3		2	2	2	2		25
pCAP-268	TPSYG**HTPSHHR**RR	WT1	5				4											9
pCAP-269	DEPLP**PPERRR**																	0
pCAP-270	SPHPPY																	0
pCAP-271	SPHPPYSPHPPYSPHPPYP																	0
pCAP-272	RRPHNLHHD																	0
pCAP-273	RDFHTIHPSISRR										3							3
pCAP-274	LRDPHPPERRIR																	0
pCAP-275	myr-MTYSDMPRRIITDEDRRR							3	3		3							9
pCAP-276	RRVDIHDGQRR				3													3
pCAP-277	DQPYPHRRIR				3													3
pCAP-278	RRYDTVIDDIEYRR				3						3							6
pCAP-279	RDTIERPEIRR				3						3							6
pCAP-280	myr-RYRRLILEIWRR			3							3							3
pCAP-281	myr-RD**FILFIRRLGR**R				3													3
pCAP-282	myr-RRPVAPDL**RHTIHIPPER**	LTA	3	3	3	3		6	6	2	2	3		4	4	3		42
pCAP-283	RRPADQISYLHPPER																	0
pCAP-284	myr-RHDTHNAHIRR							5	5							4		14
pCAP-285	RR**DIIRHNAHS**							4	4									8
pCAP-286	HDFHDYLERR							4	4									8
pCAP-287	RDFERTIVDI				4			4	4									12
pCAP-288	THDFDRLLRIRRR			2	3													5
pCAP-289	RHNHIRPDNQ			2				4	4									10
pCAP-290	RYKEPRITPRE				4			2	2									8
pCAP-291	DLQYDFPRIRR																	0
pCAP-292	YDELYQKEDPHRRR																	0
pCAP-293	RRIRIDPQHD							2										0
pCAP-294	FKPERFPQNDRRR																	0
pCAP-295	LDLYHPRERR							3										0
pCAP-296	RPADRIRR																	0
pCAP-297	HDFDPRYRDRR																	0
pCAP-298	RRIRDPLGNEHE							3										0
pCAP-299	ILQPDFLIRPE							2										0
pCAP-300	RIRRDPDSPLPHPE																	0
pCAP-301	myr-IRGRIRIIRRIR			3	3			5	5							4		20
pCAP-302	LRIEPIRIR				3			3	4									10
pCAP-303	IVEFRIRR				3													3
pCAP-304	myr-RRIRILMFLIGCGRV																	0
pCAP-305	IREFDPRRIR						4	4	4								4	16
pCAP-306	myr-RLIRIRILM			6		4												10
pCAP-307	HDPRIIRIR							2	2									4
pCAP-308	myr-**RRICRFIRICRVR**	CDC25B		6	4			6	6		3			2	3	6		36
pCAP-309	HPHVILPRIRIRIR							2	2									4
pCAP-310	RLRCLLLLIGRVGRR			4														0
pCAP-311	EIHTIHLLPERR																	0
pCAP-312	RRPRIPDYIL			2														2
pCAP-313	myr-RRREILHPEFRILYE			2				4	3									9
pCAP-314	RSTPHIHEFIRR			2														2
pCAP-315	LHFSHIDRR			3				4	4									11
pCAP-316	myr-DIHTIHLPDTHRR			3				3	3									9
pCAP-317	RRD**IHTIHPFYQ**	HSD17	4	3	4	2		5	5				5	4	4	3		39
pCAP-318	RPEFHSFHPIYERR			3				3										3
pCAP-319	SHDFYPHWMRERIR			3														3
pCAP-320	EPSHPRSRYPRTF																	0
pCAP-321	RNIIIRDFIHFSHIDR																	0
pCAP-322	RRIRDPQIK-myrLEIHFSHID																	0
pCAP-323	myr-DLHTIHIPRDRR																	0
pCAP-324	SHDFPHREPRPERR																	0
pCAP-325	myr-**RRIRDPRILLL**HFD	CCT3		3	3	4	3	5	4	4	3	4	6	4	4	6		53
pCAP-326	myr-RRHNAH**HSTPHPD**DR	RAD9A	14	3	3	4		6	6	4	2		5	4	5	4		60
